# Temporal Trends in Tuberculosis Incidence in the 1st Health Region of Alagoas, Brazil (2001–2022)

**DOI:** 10.3390/ijerph22121846

**Published:** 2025-12-10

**Authors:** Givanildo de Gois, Paulo Miguel de Bodas Terassi, Juaneza Barroso Falcão, Kelly Alonso Costa, Bruno Serafini Sobral, Marcelo Alves Muniz, Welington Kiffer de Freitas, Roberta Fernanda da Paz de Souza Paiva

**Affiliations:** 1 Institute of Atmospheric Sciences (ICAT), Federal University of Alagoas (UFAL), Av. Lourival de Melo Mota, no number—Campus A. C. Simões—Tabuleiro do Martins, Maceió 57072-970, AL, Brazil; givanildogois@gmail.com; 2 Latin American Institute of Technology, Infrastructure and Territory (ILATIT), Federal University for Latin American Integration (UNILA), 6731 Tancredo Neves Avenue—Itaipu A District, Foz do Iguaçu 85867-970, PR, Brazil; 3 Claretiano University Center, Av. Boulevard Thaumaturgo, 1180, Copacabana, Cruzeiro do Sul 69980-000, AC, Brazil; juanezaipx@hotmail.com; 4 Postgraduate Program in Environmental Technology, Fluminense Federal University (UFF), Avenue dos Trabalhadores, 420, Vila Santa Cecília District, Volta Redonda 27255-125, RJ, Brazil; kellyalonso@id.uff.br (K.A.C.); wkiffer@id.uff.br (W.K.d.F.); 5 Land and Cartography Institute of Rio de Janeiro (ITERJ), Rio de Janeiro 20060-060, RJ, Brazil; brunossobral@gmail.com; 6 Graduate Program in Environmental Sciences (PPGCA), Federal University of Acre (UFAC), Multidisciplinary Center (CMULTI), Estrada Canela Fina, km 12, Gleba Formoso, Lote 245, Colônia São Francisco, Cruzeiro do Sul 69980-000, AC, Brazil; marcelo.muniz@ufac.br; 7 Postgraduate Program in Geosciences (Geochemistry), Fluminense Federal University (UFF), Outeiro de São João Batista, Campus do Valonguinho, Mario Santos Braga Street, 30, Centro, Niterói 24020-140, RJ, Brazil; 8 Postgraduate Program in Environmental Technology, Federal Fluminense University (UFF), Technological Center, Avenue dos Trabalhadores, 420, Vila Santa Cecília District, Volta Redonda 27255-250, RJ, Brazil; robertapaz2003@yahoo.com.br

**Keywords:** public health policy, gender disparities, socioeconomic determinants, Mann–Kendall test, Pettitt test

## Abstract

The present study aimed to examine the temporal dynamics of tuberculosis incidence, mortality, and TB–HIV coinfection in the First Health Region of Alagoas from 2001 to 2022, with particular attention to sex-specific differences. The analysis revealed pronounced divergences between men and women. The male series exhibited significant positive autocorrelation and high interannual variability, indicating strong temporal dependence and heightened sensitivity to external disruptions such as the COVID-19 pandemic. The female series displayed a more regular pattern without autocorrelation. Although both sexes showed declining incidence, only the reduction among women reached statistical significance; the male trend remained unstable and inconclusive. Disease burden was consistently higher among men, who accounted for most cases and maintained incidence levels above elimination targets. TB–HIV coinfection increased in both sexes, with a sharper rise among men and a statistically significant upward trend among women, accompanied by a structural shift in 2010. Additional change points in 2014 and 2018 are likely to reflect alterations in surveillance or broader public health events. The weak performance of linear models underscores the role of persistent social determinants and inequities in healthcare access. Overall, the findings demonstrate that tuberculosis remains a major public health concern and that differentiated strategies by sex are essential for effective prevention and care.

## 1. Introduction

Tuberculosis has long constituted one of the most consequential global public health challenges. In the nineteenth century, it accounted for up to one quarter of all deaths in several European countries, with mortality rates approaching nine hundred deaths per one hundred thousand inhabitants per year in major urban centers such as London and Stockholm. Although diagnostic and therapeutic advances substantially reduced lethality, the disease remains among the principal global causes of death from transmissible infections [1,2,3,4,5,6]. Contemporary evidence indicates that persistent social inequalities, structural vulnerabilities, and obstacles to healthcare access continue to sustain transmission, particularly in lower- and middle-income settings, a pattern likewise documented in recent investigations of the spatiotemporal dynamics of tuberculosis and its drug resistance in Brazil [7,8,9,10,11,12]. The emergence of resistant lineages, combined with continued active transmission, reflects systemic fragilities and barriers to timely diagnosis and appropriate treatment, as demonstrated by [8], whose genomic and epidemiological findings reveal the coexistence of high disease burden and adverse social determinants [7].

Caused by *Mycobacterium tuberculosis* and transmitted through the airborne route, tuberculosis remains one of the most consequential infectious diseases worldwide, with approximately 9.9 million cases and 1.3 million deaths among individuals not coinfected with HIV in 2020 [13,14]. Its persistence is closely associated with socioeconomic inequalities and disproportionately affects Indigenous populations, persons deprived of liberty, individuals living with HIV or AIDS, migrants, and other socially vulnerable groups [2,3,4,15,16]. Within Latin America, Brazil stands out as the only country among the twenty-two nations designated as priorities for global tuberculosis control, concentrating eighty-two percent of regional cases and ranking seventeenth worldwide in incidence [7,10,15,17]. Marked internal heterogeneity persists. Although the Center West, South, and portions of the Northeast exhibit some of the lowest national rates, the Northeast overall sustains elevated indicators [18]. In Alagoas, the disease predominantly affects men aged forty-five to fifty-four years, with nearly twice as many cases as women, and displays pronounced spatial concentration. Seven municipalities account for roughly sixty percent of notifications, and Maceió alone represents 43.3 percent of reported cases. Despite the reduction in incidence from thirty-one to eighteen cases per one hundred thousand inhabitants between 2012 and 2021, challenges remain concerning lethality and pronounced municipal heterogeneity [14,19,20,21].

Robust statistical methods are essential for elucidating the temporal evolution of tuberculosis. Among these, the Mann–Kendall test is widely used to detect monotonic trends in time series, and the Pettitt test is valuable for identifying structural break points that may reflect the influence of public policies, modifications in surveillance systems, diagnostic changes, or epidemic shocks [22,23]. This study therefore examines tuberculosis morbidity and mortality in the First Health Region of Alagoas between 2001 and 2022, identifying trends and potential inflection points under the hypothesis that significant sex-based differences exist.

The absence of rigorous nonparametric analyses reveals an important gap in understanding temporal changes in tuberculosis incidence at subnational scales, a gap especially pertinent in socially vulnerable areas of the Brazilian Northeast. The literature predominantly focuses on spatial analyses or descriptive epidemiology, often neglecting approaches capable of capturing how interventions, epidemic disruptions, and socioenvironmental determinants shape temporal dynamics.

The administrative structure of the Unified Health System in Alagoas consists of ten Health Regions defined by demographic density, healthcare flows, epidemiological profiles, and the availability of specialized services, aiming to optimize management, planning, and the provision of healthcare. These regions comprise the following configuration, presented here in continuous prose: The First Health Region, headquartered in Maceió, includes twelve municipalities, namely Barra de Santo Antônio, Barra de São Miguel, Coqueiro Seco, Flexeiras, Marechal Deodoro, Messias, Paripueira, Pilar, Rio Largo, Santa Luzia do Norte, and Satuba. The Second Health Region encompasses nine municipalities, including Jacuípe, Maragogi, and Porto Calvo. The Third Health Region, with its seat in Murici, contains eleven municipalities such as Colônia Leopoldina and União dos Palmares. The Fourth Health Region comprises nine municipalities, including Atalaia, Capela, and Viçosa. The Fifth Health Region includes seven municipalities, such as Anadia, São Miguel dos Campos, and Teotônio Vilela. The Sixth Health Region encompasses eight municipalities, including Coruripe, Penedo, and Piaçabuçu. The Seventh Health Region, headquartered in Arapiraca, is the largest in the state and comprises seventeen municipalities, such as Batalha, Girau do Ponciano, and Limoeiro de Anadia. The Eighth Health Region includes eight municipalities, namely Belém, Palmeira dos Índios, and Tanque d’Arca. The Ninth Health Region comprises thirteen municipalities, including Canapi, Santana do Ipanema, and São José da Tapera. The Tenth Health Region consists of seven municipalities, among them Delmiro Gouveia, Mata Grande, and Piranhas.

For geographic contextualization, Brazil is a federative republic composed of twenty-six states and one Federal District, totaling twenty-seven federative units. Each state possesses administrative autonomy and its own municipal subdivision. Alagoas, situated in the Northeast Region, is subdivided into ten Health Regions for the internal organization of the Unified Health System [24].

The application of appropriate statistical tools is essential for understanding the spatiotemporal dynamics of tuberculosis and provides crucial support for public policies aimed at reducing incidence and lethality. Section 2 describes the materials and methods, including data sources, statistical procedures, and regional characterization. Section 3 presents the results with emphasis on temporal variability and the identification of inflection points. Section 4 discusses these findings considering national and international literature, emphasizing epidemiological and social determinants. Section 5 synthesizes the principal conclusions and public health implications, demonstrating how the analytical framework adopted here may guide tuberculosis control strategies in other high incidence regions.

The absence of statistical power assessment for the Mann–Kendall test compromises the interpretation of non-significant results, as this test exhibits limited sensitivity in short, highly variable, or autocorrelated time series [25]. The test often exhibits only moderate statistical power and may fail to detect weak trends when the sample size is small or when substantial noise is present in the data [26,27,28]. Consequently, a post hoc power evaluation is advisable, either through Monte Carlo simulations or through methods specifically developed for autocorrelated series [25]. Alternatively, a structured discussion of trend detectability is required, considering series length, interannual variability, and the minimum trend magnitude of epidemiological relevance. Without such analysis, results may reflect statistical insensitivity rather than the absence of an actual trend.

The present study aims to analyze the temporal dynamics of tuberculosis morbidity, mortality, and TB–HIV coinfection in the First Health Region of Alagoas from 2001 to 2022, with explicit attention to sex-specific differences. It seeks to quantify monotonic trends using the Mann–Kendall test and Sen’s slope estimator, identify potential structural change points through the Pettitt test, and compare the variability, persistence, and magnitude of epidemiological indicators between men and women. By situating these patterns within the broader socio-epidemiological context of Alagoas and the Brazilian Northeast, this study provides an analytical basis for refining surveillance, guiding public health interventions, and informing targeted tuberculosis control strategies in settings marked by structural vulnerabilities.

The article is organized as follows: Section 2 details the materials and methods, including data sources, statistical procedures, and regional characterization. Section 3 presents the results, focusing on temporal variability and the identification of change points. Section 4 discusses these findings in comparison with national and international literature, emphasizing epidemiological and social determinants. Finally, Section 5 summarizes the main conclusions and public health implications, highlighting how the analytical framework adopted here can inform targeted TB control strategies in other high-burden regions.

### Study Area

**Figure 1:**
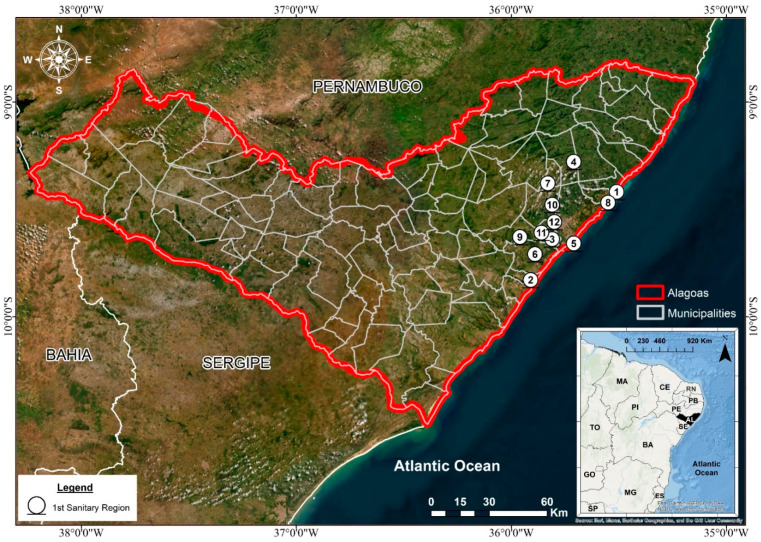
Distribution of the municipalities in the 1st Health Region of the state of Alagoas. Source: Authors, 2025.

The present study was conducted in the 1st Health Region of the State of Alagoas (1st HRA), located in Northeastern Brazil. This administrative region encompasses twelve municipalities: Maceió, Rio Largo, Pilar, Satuba, Santa Luzia do Norte, Coqueiro Seco, Marechal Deodoro, Barra de São Miguel, Paripueira, Barra de Santo Antônio, Messias, and Flexeiras (Table 1; Figure 1). Collectively, these municipalities form a heterogeneous area that integrates coastal, urban, peri-urban, and rural environments, reflecting distinct socioeconomic and demographic profiles. The inclusion of the state capital, Maceió, which concentrates a large share of the regional population and health infrastructure, alongside smaller and less-developed municipalities, allows for a comprehensive assessment of how social and spatial inequalities influence the temporal dynamics of tuberculosis (TB) incidence in the region.

Alagoas is the second smallest state in the Northeastern Region of Brazil (RNEB), covering an area of 27,830.661 km^2^. According to the Brazilian Institute of Geography and Statistics [29], the state has an estimated population of 3,127,683 inhabitants, distributed across 102 municipalities, with an average population density of 112.38 inhabitants per km^2^. This figure masks strong internal contrasts, as population density is considerably higher in the coastal and metropolitan zones—especially in Maceió—compared to the interior municipalities, where rural settlement patterns and lower population densities prevail. These demographic differences are directly related to disparities in infrastructure, sanitation, education, and healthcare access, which in turn influence the spatial distribution and persistence of TB across the state.

From a socioeconomic perspective, Alagoas consistently ranks among the most socially vulnerable states in Brazil. It presents the lowest Municipal Human Development Index (MHDI) nationwide, with a mean value of 0.684 [29], which remains below the national average (0.760). Within the 1st HRA, municipalities such as Maceió, Rio Largo, Marechal Deodoro, Pilar, Paripueira, and Satuba exhibit moderate human development levels (MHDI ≥ 0.600), while Flexeiras, Barra de São Miguel, Messias, Coqueiro Seco, and Santa Luzia do Norte record lower values (MHDI ≤ 0.600). These marked inequalities underscore persistent structural challenges—such as income concentration, housing deficits, and limited access to public services—that are recognized determinants of TB incidence and treatment outcomes [2,4,16].

Geographically, the 1st HRA occupies a strategic position along the eastern coastal zone of Alagoas, integrating both densely urbanized municipalities and rural localities with fragile health networks. Its physiographic characteristics include low altitudes, humid tropical climate, and a combination of coastal plains and tabuleiros (plateaus), which sustain a high concentration of population and mobility corridors linking Maceió to other municipalities in the state. These features contribute to both disease dissemination and the complexity of TB surveillance, reinforcing the need for region-specific epidemiological analyses capable of identifying temporal fluctuations and structural changes in disease pattern.

Therefore, the selection of the 1st HRA as the spatial focus of this research was motivated by its epidemiological relevance, demographic concentration, and pronounced social contrasts. This territorial framework allows for a detailed evaluation of how local socioeconomic and environmental conditions interact with public health determinants to shape TB morbidity and mortality trends.

## 2. Materials and Methods

### 2.1. Study Design and Setting

This investigation was designed as an ecological, quantitative, and exploratory study, with emphasis on the temporal pattern of morbidity and mortality indicators for tuberculosis in the state of Alagoas, located in the Northeast Region of Brazil, from 2001 to 2022. The adoption of an ecological design was grounded in the use of population-level aggregated data derived from a compulsory notification system, which enables the assessment of long-term population patterns and temporal trends that are pertinent to public health planning. Such an approach is particularly suitable for examining diseases shaped by strong social determinants and marked regional disparities, as it facilitates the identification of epidemiological patterns that extend beyond individual-level variability [30,31]. This methodological orientation has been widely recommended in contemporary studies of tuberculosis epidemiological surveillance, including recent analyses published in *Pathogens* [8].

The methodological structure of this study was organized into two sequential analytical stages. In the first stage, the statistical characteristics of the tuberculosis time series were examined to ensure the validity of the subsequent trend analyses. Specifically, the Shapiro–Wilk test was employed to assess the normality of the data, determining whether the distribution of annual case counts satisfied the assumptions required for parametric procedures. This test is particularly valuable in epidemiological research, where deviations from normality are common because of reporting inconsistencies, seasonal fluctuations, or the effects of policy interventions [31]. In parallel, the Bartlett test was applied to evaluate the homogeneity of variances across the study period, a critical requirement for ensuring the reliability of comparative inferences over time [30].

Subsequently, two annual time series (2001–2022) corresponding to the percentage rates of tuberculosis in the male and female populations were analyzed. For each series, a simple linear regression model was fitted, treating the rates as the dependent variable and the year as the explanatory variable, with the objective of identifying linear trends throughout the period.

The independence of residuals was evaluated using the Durbin–Watson test, which yields values ranging from zero to four, where results below two indicate positive autocorrelation, values above two denote negative autocorrelation, and values approximating two suggest the absence of first-order autocorrelation.

At this stage, a descriptive and exploratory analysis was also conducted, encompassing measures of central tendency and dispersion for newly reported tuberculosis cases. These data were obtained from the Notifiable Diseases Information System (SINAN), accessed through the open-access database [32] maintained by the Brazilian Ministry of Health. The descriptive analysis provided an initial epidemiological characterization of the tuberculosis burden in Alagoas and supported the subsequent inferential modeling.

The second stage focused on identifying monotonic temporal trends and abrupt structural changes in tuberculosis incidence, disaggregated by sex. For this purpose, the nonparametric Mann–Kendall test was applied, given its robustness in detecting consistent increasing or decreasing trends in time series that do not satisfy assumptions of normality or linearity [25,26]. This test is widely recognized in environmental and health sciences for its capacity to accommodate long-term, irregular, and heterogeneous data, conditions frequently observed in surveillance systems characterized by underreporting or policy-driven discontinuities [8].

Complementarily, the Pettitt test [23] was employed to detect abrupt change points (breakpoints) within the temporal sequence. This nonparametric method is particularly effective for identifying moments of structural shift in epidemiological time series, such as those arising from modifications in public health policies, vaccination coverage, diagnostic technologies, or case notification protocols. Its inclusion in the analytical framework allows for the identification of critical inflection years in TB incidence, potentially corresponding to transitions in surveillance capacity or broader social and economic changes affecting disease transmission dynamics.

All analyses were performed using the R statistical environment [33], which offers a versatile platform for reproducible epidemiological analysis and includes specialized packages for nonparametric trend testing and change-point detection. The use of open-source software (R, version 4.4.2; R Foundation for Statistical Computing, Vienna, Austria) enhances transparency and replicability—key principles for the integrity of population health research.

In summary, this study follows a three-phase methodological sequence:

(1) Data structuring and preliminary validation, involving verification of normality and variance homogeneity to define the appropriate statistical approach;
(2) Descriptive and exploratory analysis of temporal patterns in TB incidence, providing a baseline epidemiological overview; and
(3) Inferential modeling through the Mann–Kendall and Pettitt tests to identify both monotonic trends and abrupt structural changes in the time series, disaggregated by sex.

This comprehensive analytical framework provides a robust foundation for interpreting temporal dynamics in TB morbidity and supports the development of evidence-based public health strategies in socially vulnerable regions such as Alagoas.

### 2.2. Control, Structuring, and Quality Assurance of Tuberculosis Notification Data

Initially, the monthly notification data for newly reported tuberculosis cases were organized in a spreadsheet and subjected to a preliminary quality control procedure aimed at identifying potential errors or inconsistencies within the time series. Following this verification, it was determined that gaps or interruptions were present in the monthly data for the 2001–2022 period. The percentages of missing entries were computed through the application of the mstats function using R software, version 3.4.5 [33] (R Core Team, 2025). Further methodological details are provided in [34].

### 2.3. Data Acquisition and Descriptive–Exploratory Analysis

The data analyzed in this study comprised confirmed and notified cases of tuberculosis (TB) recorded in the state of Alagoas between 2001 and 2022, obtained from the Information System for Notifiable Diseases (SINAN). These data are publicly available through the Department of Informatics of the Brazilian Unified Health System (DATASUS), an open-access national database managed by the Ministry of Health of Brazil [32]. The use of SINAN–DATASUS data ensures methodological transparency and comparability, given its standardization across Brazilian municipalities and its relevance for longitudinal epidemiological surveillance.

Initially, the TB notification data were subjected to statistical validation procedures to verify the assumptions of normality and homogeneity of variance in the monthly time series for both male and female populations. The Shapiro–Wilk test was applied to assess whether the data conformed to a normal distribution, an essential step for determining the appropriateness of subsequent statistical methods. Testing for normality is particularly relevant in epidemiological time series, as deviations frequently occur due to irregular reporting, policy shifts, or seasonal variation in disease incidence [31]. Complementarily, the Bartlett test was employed to evaluate the homogeneity of variances, ensuring that variance equality assumptions were met across the temporal dataset—an important condition for reliable inferential analysis [30].

Following these preliminary tests, a comprehensive descriptive and exploratory statistical analysis was performed to characterize the temporal pattern of TB incidence within the 1st Health Region of Alagoas (1st HRA). The analysis included measures of central tendency (mean, median), dispersion (standard deviation, range, and coefficient of variation), and extreme values (minimum and maximum). In addition, boxplot visualizations were generated to graphically represent the monthly variability and outlier distribution of new TB cases by sex. This exploratory phase provided a detailed overview of the dataset’s structure, variability, and potential asymmetries, supporting the interpretation of subsequent inferential tests.

All data processing and statistical computations were conducted using the R statistical software, version 4.3.0 [33]. The use of R ensured analytical reproducibility and allowed for the application of specialized packages for nonparametric trend detection and change-point analysis, in accordance with current best practices in epidemiological research.

### 2.4. Assessment of Statistical Assumptions and Analytical Procedures

The verification of normality hypotheses ($H_{0}$ and $H_{1}$) for the monthly time series of new tuberculosis (TB) cases, disaggregated by sex (male and female), was carried out using the parametric Shapiro–Wilk test [35] at a 5% significance level (α=0.05), following the recommendations of [36]. This test evaluates whether the data distribution approximates a normal curve, which is crucial for determining the adequacy of parametric or nonparametric analyses.

The Shapiro–Wilk test statistic (W) is defined as follows:

(1) $$W=\frac{{\left[\sum_{i=1}^{k}a_{n-i+1}\left(y_{n-i+1}-y_{i}\right)\right]}^{2}}{\sum_{i=1}^{n}{\left(y_{i}-\bar{y}\right)}^{2}}\;=\;\frac{{\left[\sum_{i=1}^{k}a_{i\;}y_{\left(i\right)}\right]}^{2}}{\sum_{i=1}^{n}{\left(y_{i}-\bar{y}\right)}^{2}}$$

Considering X as the variable under study, the following hypotheses were formulated:

**H_0_:**  *The new TB cases are normally distributed in the 1st Health Region of Alagoas (1st HRA).*

**H_1_:**  *The new TB cases are not normally distributed in the 1st Health Region of Alagoas (1st HRA).*

The conditions for the dataset to exhibit a normal distribution at the probability level α, based on the assumptions, are as follows:

- For $W_{cal}\leq W_{tab}$, reject $H_{0}$ when p≤0.05 (Significant—S);
- For $W_{cal}\geq W_{tab}$, accept $H_{0}$ when p≥0.05  (Not Significant—NS).

The hypothesis of variance homogeneity for new TB cases was assessed using the Bartlett test [37], following the methodology proposed by [30]. This test evaluates the assumption that *k* independent samples drawn from a population exhibit equal variances, that is, homogeneity of variances. The Bartlett test statistic (*B*_0_) is determined according to the following Equation (2).

(2) $$B_{0}=\frac{ln\sum_{i=1}^{k}\left(n_{i}-1\right)\sum_{j=1}^{n_{i}}\frac{{\left(y_{ij}-\bar{y_{i}}\right)}^{2}}{n_{i}-1}-\sum_{i=1}^{k}\left[\left(n_{i}-1\right)ln\sum_{j=1}^{n_{i}}\frac{{\left(y_{ij}-\bar{y_{i}}\right)}^{2}}{n_{i}-1}\right]}{1+\frac{1}{3\left(k-1\right)}\left(\sum_{i=1}^{n}\frac{1}{{\;n}_{i}-1}-\frac{1}{\sum_{j=1}^{n}n_{j}-k}\right)}$$

Regarding the hypothesis:

Where: *N* represents the total number of observations, *nⱼ* and *k* denote the number of observations within each group, χ^2^ corresponds to the chi-square distribution, and *B*_0_ represents the Bartlett test statistic.

Considering *X* as the variable under analysis, the following hypotheses were formulated:

**H_0_:**  *The new TB cases exhibit homogeneous variances (not significant—NS).*

**H_1_:**  *The new TB cases do not exhibit homogeneous variances (significant—S).*

The decision criteria for determining the homogeneity or heterogeneity of variances at a given probability level (*α*) were established as follows:

- Reject H_0_ when *p*-value ≤ 0.05;
- Fail to reject H_0_ when *p*-value ≥ 0.05.

Two annual time series (2001–2022) corresponding to the percentage rates of tuberculosis in the male and female populations were analyzed, and for each series a simple linear regression model was fitted, with the rates treated as the dependent variable and the year as the explanatory variable. The verification of first-order autocorrelation in the residuals was performed using the Durbin–Watson test, whose statistic ranges from zero to four, where values below two indicate positive autocorrelation, values above two indicate negative autocorrelation, and values near two denote the absence of autocorrelation. Statistical significance was assessed at the five percent level, and all analyses were conducted in R software using the lmtest package.

The proportion of coinfection (%TB/sex) and the incidence rate (TITB/sex) of TB cases were calculated annually in this study, following the methodology established by the Department of Epidemiological Surveillance of the Brazilian Ministry of Health (MS). These indicators were derived based on the total number of new TB cases and their distribution by sex. The calculations were expressed through Equations (S1) and (S2) as follows. Equation (S1) provides the formal expression for estimating the proportion of tuberculosis cases by sex (%TB/sex). Subsequently, the sex-specific tuberculosis incidence rates (TITB/sex) were calculated according to Equation (S2).

The analysis of potential trends in the time series of tuberculosis (TB) data was based on the application of the nonparametric Mann–Kendall trend test (MKT), following the methodology proposed by [22,38] and is used in research in the fields of Climatology and Health Geography by [39,40,41,42,43].

However, as highlighted by several authors, the MKT should be specifically used to detect monotonic trends. According to [40,44], the MKT is a robust tool for testing the null hypothesis (H_0_) of the absence of a monotonic trend within a time series.

Numerous studies have emphasized the importance of the MKT in various fields of health sciences, particularly in analyzing the temporal pattern of epidemiological indicators, as reported by [43,45,46,47]. Furthermore, as discussed by [40,48], when a time series (*x*_1_, *x*_2_, …, *x_n_*) consists of *n* independent and identically distributed random variables, the Mann–Kendall statistic (*S*) is expressed as shown in Equation (3):

(3) $$S=\sum_{i=1}^{n-1}\sum_{j=i+1}^{n}sign\left(x_{j}-x_{i}\right)$$

where: *n* represents the total number of observations, and *xᵢ* and *xⱼ* correspond to the values of the observations at times *i* and *j* (*j* > *i*).

The sign function associated with the Mann–Kendall statistic is formally defined in Equation (S3) (Sign Function for the Mann–Kendall Test). The statistic *S* is asymptotically normally distributed under the null hypothesis, with its expected value specified in Equation (S4) (Expected Value of the Mann–Kendall Statistic) and its variance presented in Equation (S5) (Variance of the Mann–Kendall Statistic). In the presence of tied observations, the variance is adjusted following Equation (S6) (Tie-Adjusted Variance of the Mann–Kendall Statistic). The statistical significance of the Mann–Kendall test is evaluated through a two-tailed procedure based on the standardized Z-statistic, whose formulation is presented in Equation (S7) (Standardized Z-Statistic for the Mann–Kendall Test).

The null hypothesis (H_0_) states that there is no trend in the time series, and it is rejected when $|Z_{MK}|>Z_{\alpha /2}$, where *α* denotes the significance level and *Z_{α/2}* is the corresponding critical value of the standard normal distribution at a probability of *α/2*. The sign of *Z_mk_* indicates the direction of the trend: positive values represent an increasing trend, whereas negative values indicate a decreasing trend.

The *p*-value associated with statistic *S* is obtained from the cumulative probability of the standard normal distribution. According to [18], the MKT is a sequential, nonparametric method designed to verify whether a time series exhibits a statistically significant monotonic trend, leading to the acceptance or rejection of the null hypothesis (H_0_) of no positive or negative trends, at a 5% significance level (*α* = *0.05*), as adopted in this study.

The hypotheses formulated in this study are as follows:

**H_0_:**  *The TB case observations in the 1st Health Region of Alagoas are independent and identically distributed (no trend).*

**H_1_:**  *The TB case observations in the 1st Health Region of Alagoas are not independent and identically distributed (presence of trend).*

Accordingly, the statistical significance of the Mann–Kendall test was interpreted as follows:

- *p*-value ≥ *α* → Fail to reject H_0_;
- *p*-value ≤ *α* → Reject H_0_.

The Mann–Kendall test (MKT) is recognized in the literature as a robust method for detecting trends in time series data. However, it does not provide the magnitude of the identified trend. According to [18], this magnitude can be estimated using the Sen’s slope estimator (*Sen* = *Q_ij_* = *median*), as expressed in Equation (4):

(4) $$Sen\;=\;median(Q_{ij})\;=\;(X_{j}\;-\;X_{i})/(j\;-\;i),\;\;\;\;\;\;for\;i\;<\;j$$

where: *X_i_* and *X_j_* represent the values of the variable under analysis in years *i* and *j*, respectively. A positive or negative value of *Q* indicates an increasing or decreasing trend. If a time series comprises *n* observations, the total number of estimated *Q* pairs for the Sen slope estimator is determined according to Equation (S8).

In the present study, the following hypotheses were formulated:

**H_0_:**  *The TB case observations in the 1st Health Region of Alagoas (1st HRA) are independent and identically distributed (no trend).*

**H_1_:**  *The TB case observations in the 1st Health Region of Alagoas (1st HRA) are not independent and identically distributed (presence of trend).*

The Pettitt Test [33] was employed to detect potential shifts in TB data within the 1st Health Region of Alagoas. The null and alternative hypotheses (H_0_ and H_1_) were defined as follows:

**H_0_:**  *There is no abrupt change in the time series.*

**H_1_:**  *There is an abrupt change point in the time series.*

The statistical significance of the test was determined as follows:

- *p*-value ≥ *α* → Fail to reject H_0_.
- *p*-value ≤ *α* → Reject H_0_.

The U_t,t_ statistic of the test measures how many times an element from the first sample is greater than one from the second, as shown in Equation (5):

(5) U_tT=U_(t−1,T)+Σ_(j=1)^Tsgn(X_i−X_j),    for t=2,…,T

where: sgn (x) = 1 for x > 0; sgn (x) = 0 for x = 0; and sgn (x) = −1 for x < 0. The statistic U_t,t_ is calculated for values of 1 < t < T, and the k (t) statistic of the PT is defined in Equation (S9).

This statistic identifies the point at which an abrupt shift occurs in the mean of the TB time series, and its significance can be approximately estimated by Equation (6):

(6) $$p\equiv 2exp\left\{-6k{\left(t\right)}^{2}/\left(T^{3}+T^{2}\right)\right\}$$

The abrupt change point in the series corresponds to the value of *t* at which *k*(*t*) reaches its maximum. The critical *K* values can be determined using Equation (7):

(7) $$K_{crit}=\mp \;\sqrt{\frac{-ln\left(p/2\right)/\left(T^{3}+T^{2}\right)}{6}}$$

### 2.5. Considerations on Statistical Power and Sample Robustness

To assess the presence of temporal trends in the annual tuberculosis rates, the nonparametric Mann–Kendall and Pettitt tests were employed, both of which are widely used in epidemiological time-series analyses due to their robustness and their independence from strict normality assumptions. The selection of these methods reflects the nature of surveillance data, which are frequently characterized by annual variability, potential asymmetries, and the possibility of structural discontinuities. The analyses were conducted using two annual time series (2001–2022) corresponding to the percentage rates of tuberculosis in the male and female populations. For each series, a simple linear regression model was also fitted, treating the annual rates as the dependent variable and the year of notification as the explanatory variable, thereby complementing the trend assessment yielded by the nonparametric tests.

### 2.6. Evaluation of Statistical Power and Sample Limitations

The dataset consists of twenty-two observations (*n* = 22 years), which provides adequate statistical power for detecting moderate to pronounced trends, such as the reduction observed from thirty-one to eighteen cases per one hundred thousand inhabitants over the study period. Nevertheless, it is acknowledged that time series of this length may exhibit limited sensitivity for detecting subtle shifts, gradual declines, or inflection points of small magnitude. The intrinsic variability of surveillance data, including annual fluctuations, modifications in reporting systems, and external influences such as the COVID-19 pandemic, may introduce statistical noise capable of affecting the performance of the tests, particularly the Pettitt test, in identifying discrete structural breaks. These considerations informed us of a cautious interpretation of the results.

### 2.7. Future Analytical Directions

In recognition of these limitations, future studies may benefit from complementary methodological approaches, including longer time series, segmented regression models, Bayesian frameworks, or smoothing techniques that may enhance sensitivity to gradual changes. Stratified analyses by age group, TB-HIV coinfection status, or geographic area, as well as the incorporation of socioeconomic indicators and measures of healthcare-service coverage, may further strengthen the interpretive robustness of the observed temporal trends.

## 3. Results

**Figure 2:**
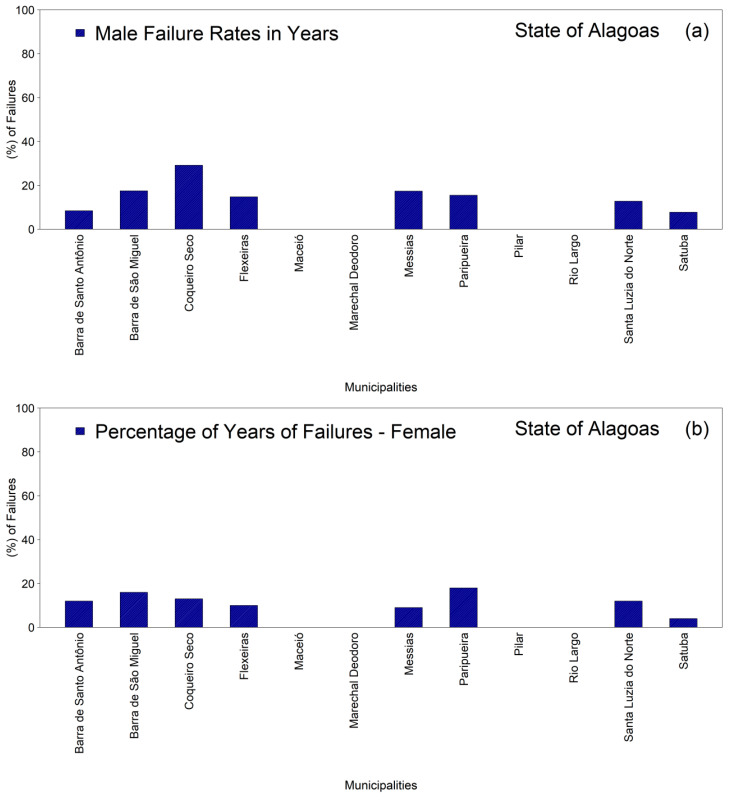
Distribution of the percentages of missing monthly tuberculosis case-notification data in the municipalities comprising the First Health Region of Alagoas (1st HRA), 2001–2022: (**a**) male population; (**b**) female population. Source: Authors, 2025.

The analysis of the percentages of missing data in the time series revealed marked heterogeneity among the municipalities of the First Health Region of Alagoas in both the male and female groups (Figure 2a,b). Among men (Figure 2a), the highest proportions of missing data were observed in Coqueiro Seco, approximately thirty percent, followed by Barra de São Miguel and Messias, both approximately eighteen percent, and Paripueira, approximately sixteen percent. Intermediate values were recorded in Flexeiras, approximately fifteen percent, Santa Luzia do Norte, approximately thirteen percent, and Barra de Santo Antônio, approximately nine percent, whereas Satuba exhibited the lowest percentage, approximately eight percent.

In the female group (Figure 2b), although the variability was more limited, Paripueira exhibited the highest proportion of missing data, approximately eighteen percent, followed by Barra de São Miguel, approximately sixteen percent, Coqueiro Seco, approximately fourteen percent, Santa Luzia do Norte, approximately thirteen percent, Barra de Santo Antônio, approximately twelve percent, and Flexeiras, approximately eleven percent. The lowest proportions were observed in Messias, approximately ten percent, and Satuba, approximately six percent. These findings indicate that, although most municipalities presented moderate levels of missing information, certain areas exhibited more pronounced weaknesses in the continuity of their time series, thereby underscoring the need to strengthen local procedures for data recording and epidemiological surveillance.

**Figure 3:**
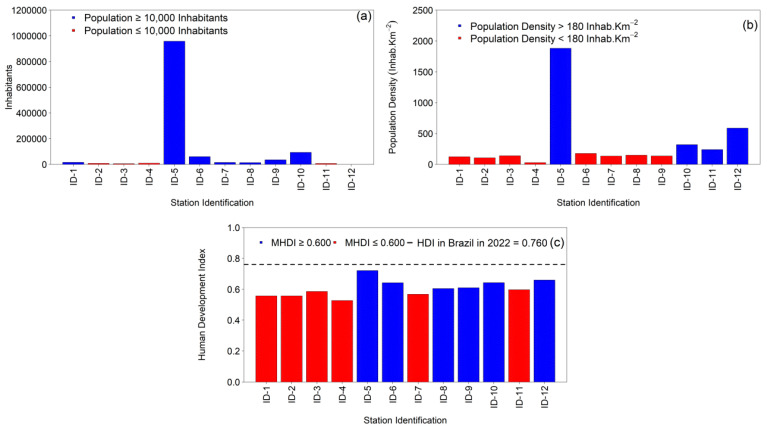
Geographic and socioeconomic characteristics of the twelve municipalities comprising the 1st Health Region of Alagoas (1st HRA) from 2001 to 2022: (**a**) total population; (**b**) population density (inhabitants·km^−2^); and (**c**) Municipal Human Development Index (MHDI), with the dashed line indicating the Brazilian national average for 2022 (HDI = 0.760). Source: Authors, 2025.

The 1st Health Region of Alagoas (1st HRA) exhibits marked socio-demographic heterogeneity (Table 1; Figure 3a–c). A metropolitan core—Maceió—dominates the regional structure, concentrating 78.31% of the total population (957,916 inhabitants) and the highest population density (1880.77 inhabitants·km^−2^) while also presenting the highest Human Development Index (HDI) in the region (0.721). A second stratum, with intermediate population shares and densities, includes Rio Largo (7.678%; 93,927 inhabitants; 319.68 inhabitants·km^−2^), Satuba (24,278 inhabitants; 588.30 inhabitants·km^−2^), Marechal Deodoro (4.935%; 60,370 inhabitants; 177.05 inhabitants·km^−2^), Pilar (2.891%; 35,370 inhabitants; 136.24 inhabitants·km^−2^), and Paripueira and Messias (each near 1.2% of the regional population with densities of 149.10 and 134.95 inhabitants·km^−2^, respectively). A third cluster—small-population municipalities (≤1%)—comprises Barra de São Miguel (0.649%; 7944 inhabitants; 106.99 inhabitants·km^−2^), Coqueiro Seco (0.456%; 5581 inhabitants; 140.91 inhabitants·km^−2^), Flexeiras (0.786%; 9618 inhabitants; the lowest density, 28.82 inhabitants·km^−2^), and Santa Luzia do Norte (0.566%; 6919 inhabitants; 239.77 inhabitants·km^−2^). Although Satuba contributes a very small share to the regional total (0.002%), it is characterized by high density (588.30 inhabitants·km^−2^).

From a social standpoint (Figure 3c), six municipalities fall within medium development (HDI ≥ 0.600): Maceió (0.721), Satuba (0.660), Rio Largo (0.643), Marechal Deodoro (0.642), Pilar (0.610), and Paripueira (0.605). The remaining five municipalities register HDI ≤ 0.600—Flexeiras (0.527), Barra de São Miguel (0.557), Messias (0.568), Coqueiro Seco (0.586), and Santa Luzia do Norte (0.597). These values contrast with Brazil’s national HDI in 2022 (0.760) and are consistent with the position of Alagoas as the lowest-HDI federation unit in the country (0.684) (IBGE, 2024). The resulting socio-spatial gradient is likely to modulate tuberculosis (TB) transmission dynamics across the 1st HRA (Table 1; Figure 3).

**Figure 4:**
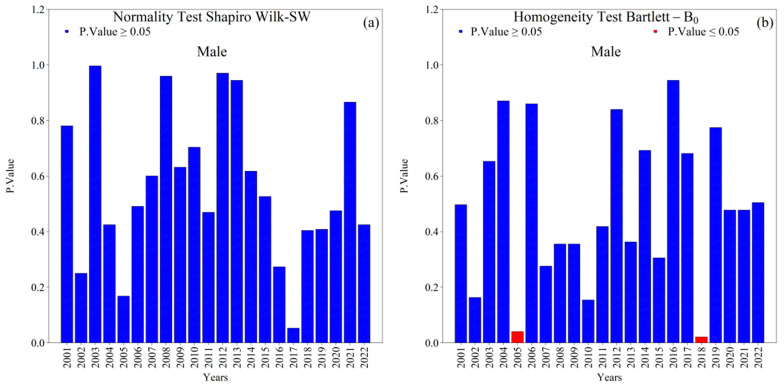
Normality (Shapiro–Wilk) and homogeneity of variance (Bartlett) tests for annual new male tuberculosis (TB) cases in the 1st Health Region of Alagoas, covering the period from 2001 to 2022: (**a**) Shapiro–Wilk normality test and (**b**) Bartlett homogeneity of variance test, with blue bars indicating non-rejection of the null hypothesis (*p* ≥ 0.05) and red bars indicating rejection of the null hypothesis (*p* ≤ 0.05). Source: Authors, 2025.

**Figure 5:**
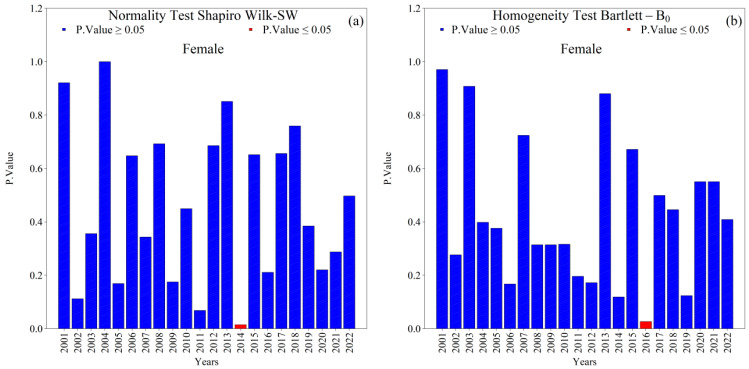
Normality (Shapiro–Wilk) and homogeneity of variance (Bartlett) tests for annual new female tuberculosis (TB) cases in the 1st Health Region of Alagoas, covering the period from 2001 to 2022: (**a**) Shapiro–Wilk normality test and (**b**) Bartlett homogeneity of variance test, with blue bars indicating non-rejection of the null hypothesis (*p* ≥ 0.05) and red bars indicating rejection of the null hypothesis (*p* ≤ 0.05). Source: Authors, 2025.

Overall, the Shapiro–Wilk (SW) normality and Bartlett (*B*_0_) homogeneity tests indicated that annual new TB cases (male and female) in the 1st HRA were normally distributed with homogeneous variances (*p*-value ≥ 0.05) across the 2001–2022 series (Figure 4 and Figure 5). Exceptions were notable and analytically relevant: female cases in 2014 violated normality (SW, *p*-value ≤ 0.05; Figure 5a), and variance heterogeneity occurred in 2005, 2016, and 2018 (Bartlett, *p*-value ≤ 0.05; Figure 4b and Figure 5b). These deviations suggest year-specific perturbations (e.g., reporting practices, service coverage, diagnostic shifts) that warrant attention in the interpretation of temporal changes.

**Figure 6:**
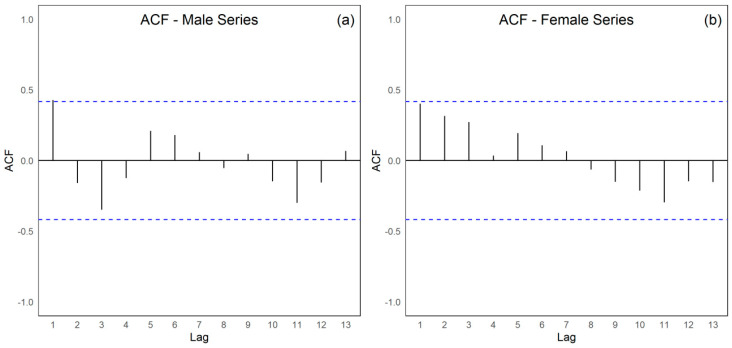
Autocorrelation function of tuberculosis (TB) case series from 2001 to 2022 in the First Health Region of Alagoas: (**a**) male population; and (**b**) female population, with dashed lines representing the approximate 95% confidence limits (±1.96/√n). Source: Authors, 2025.

The integrated assessment of the male (PCTB_MASC) and female (PCTB_FEMILE) tuberculosis time series (Figure 6a,b) from 2001 to 2022 discloses pronounced divergences in their temporal dynamics, directional tendencies, and the statistical adequacy of the models applied. The male series exhibits statistically significant positive autocorrelation, as evidenced by the Durbin–Watson statistic (DW = 1.178; *p* = 0.01034) and by the configuration of the autocorrelation function, which demonstrates persistent serial dependence indicative of substantial interannual continuity. In contrast, the female series reveals no significant autocorrelation (DW = 1.6692; *p* = 0.1488), suggesting a temporal pattern characterized by greater stochastic independence and minimal influence of preceding observations. Although both series display an overall downward trajectory, the magnitude of this decline diverges considerably. The male series presents a negative Sen’s slope of −0.0209, which is non-significant (*p* = 0.2251), whereas the female series demonstrates a statistically significant negative Sen’s slope of −0.0445 (*p* = 0.0061), indicating a more pronounced and sustained reduction over the period examined. The rate of decline in the female series is nearly twice that observed in the male series.

The presence of serial autocorrelation in the male series renders classical linear regression models inappropriate in the absence of corrective procedures, thereby necessitating the application of techniques specifically suited to correlated error structures, such as the Prais–Winsten or Cochrane–Orcutt estimators, autoregressive integrated moving-average (ARIMA) frameworks, or Newey–West heteroskedasticity and autocorrelation-consistent error adjustments. Conversely, for the female series, conventional linear modeling remains statistically permissible. From an epidemiological standpoint, the pronounced temporal persistence observed among men may reflect heightened exposure to adverse social determinants, greater structural vulnerability, or more continuous transmission pathways, whereas the more substantial and consistent decline observed among women may be attributable to higher adherence to treatment regimens, improved access to healthcare services, or greater responsiveness to preventive and early detection strategies.

The assessment of residual independence constitutes a fundamental step in validating regression models applied to time series. In this study, the Durbin–Watson test was applied to both the male (PCTB_MALE) and female (PCTB_FEMALE) series to determine the presence of first-order serial autocorrelation (Table 2). The test produced a Durbin–Watson statistic of 1.178 with a *p*-value of 0.01034, under the alternative hypothesis of positive autocorrelation. This value, being markedly below two, indicates a tendency toward positive autocorrelation in the residuals. The statistical significance reflected in the *p*-value below the five percent threshold confirms that such autocorrelation is genuine rather than random.

These findings demonstrate that the simple linear model fitted to the male series fails to satisfy the assumption of independence of errors, revealing significant temporal dependence. Consequently, the model cannot be regarded as fully adequate without the application of corrective procedures. Considering this violation, the analysis warrants methodological approaches specifically designed to address serial autocorrelation, which may include generalized least squares estimators such as the Prais–Winsten or Cochrane–Orcutt methods for autocorrelation of order one, the use of Newey–West robust standard errors, the explicit modeling of dependence through ARIMA frameworks, or, in cases where the primary objective is the identification of temporal trends, the application of nonparametric procedures such as the Mann–Kendall test and Sen’s slope estimator.

For the female series (PCTB_FEMALE), the test yielded a Durbin–Watson statistic of 1.6692 with a *p*-value of 0.1488. Although the statistics are also below two, which may visually suggest the possibility of positive autocorrelation, the *p*-value exceeding the five percent threshold indicates that there is no sufficient statistical evidence to reject the hypothesis of independence of the residuals. Accordingly, there is no significant autocorrelation in the female series, and the simple linear model may therefore be considered appropriate with respect to the assumption of error independence. The comparative analysis of annual tuberculosis coefficients between men and women in the First Health Region of Alagoas reveals distinct epidemiological patterns in both magnitude and temporal variability. The male series exhibits greater structural instability, wider dispersion, and a higher frequency of atypical years, whereas the female series displays a more regular temporal pattern with moderate fluctuations and narrower distributional amplitudes.

Between 2001 and 2022, a total of 14,197 new tuberculosis cases were reported in the First Hospital Risk Assessment system (DATASUS), with clear male predominance: 8861 cases, corresponding to 62.41 percent, were recorded among men, in contrast to 5336 cases, or 37.59 percent, among women (Tables S1 and S2). Within the male group, several years exhibited pronounced instability, notably 2002, 2003, 2004, and 2020, all of which registered coefficients of variation exceeding thirty percent, elevated interquartile ranges, and exceptionally wide extreme limits, including negative lower bounds in 2020. The recurrent presence of positive skewness and platykurtic distributions reflects flatter and more dispersed profiles, with markedly high maximum values in years such as 2002 (maximum = 63) and 2004 (maximum = 53). These results indicate heightened intra-annual heterogeneity and increased sensitivity to external events, as evidenced by the sharply irregular pattern observed in 2020, likely associated with the impact of the COVID-19 pandemic on detection and notification processes.

By contrast, the female series demonstrates smaller annual amplitudes, reduced variability, and fewer structurally atypical years. Most years exhibit coefficients of variation between sixteen and thirty two percent, with moderate peaks in 2003 (33.13 percent), 2005 (29.79 percent), and 2020 (36.24 percent). Although 2020 also represents the principal inflection point within the female series, its instability is less pronounced than that observed among men. The interquartile ranges for the female group are consistently smaller, predominantly between 3.25 and 8.00, indicating narrower concentrations around the median. Furthermore, the annual maximum values in the female group remain consistently lower, generally between twenty-eight and thirty-eight, and both lower and upper bounds follow more stable patterns, without negative lower limits.

Another salient difference is that the female series frequently presents means and medians in proximity, often practically aligned (2001, 2002, 2004, 2012), suggesting distributions that are less skewed and more homogeneous. Although positive skewness occurs in most years, its magnitude is smaller than that observed in the male series, and kurtosis remains predominantly platykurtic, reinforcing the presence of smoother dispersions without extreme events.

Overall, the comparison demonstrates that the male population concentrates the highest values, the greatest dispersion, and the most pronounced temporal instability, constituting the series most susceptible to structural breaks, abrupt peaks, and extensive intra-annual variation. The female series, in contrast, reveals a more stable pattern with lower magnitude, reduced variability, and substantially more regular pattern across the two decades examined. These differences suggest that the male component of the regional epidemic is more volatile and more strongly shaped by behavioral, social, and operational factors, whereas the female series reflects a more gradual dynamic that is less prone to extreme oscillations, thereby requiring differentiated approaches to statistical modeling, epidemiological surveillance, and control strategies.

**Figure 7:**
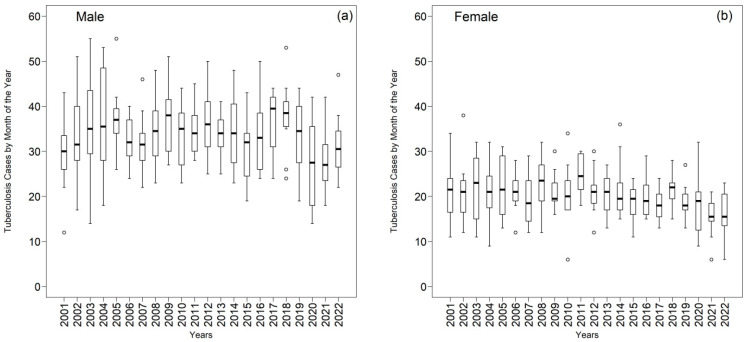
Annual distribution of tuberculosis (TB) cases by month of the year in the First Health Region of Alagoas from 2001 to 2022: (**a**) male population and (**b**) female population. In each boxplot, the median is indicated by the central line, the box represents the interquartile range (Q1–Q3), the whiskers correspond to values within 1.5 times the interquartile range, and the circles denote outliers. Source: Authors, 2025.

Boxplots provide a compact view of dispersion and central tendency (Figure 7). Among men, means and medians were ≥30 cases in 81.82% and 86.36% of years, respectively; the series peaked in 2017–2018 (mean 39.50, median 37.75) and dipped in 2020–2021 (mean 26.83, median 27.00), consistent with possible COVID-19–related under-notification and care disruptions. Among women, 63.64% of means and 50% of medians were >20 cases; 2011 showed the highest central tendency (mean 24.92, median 24.50), whereas 2021–2022 recorded the lowest values (mean/median 15.50).

**Figure 8:**
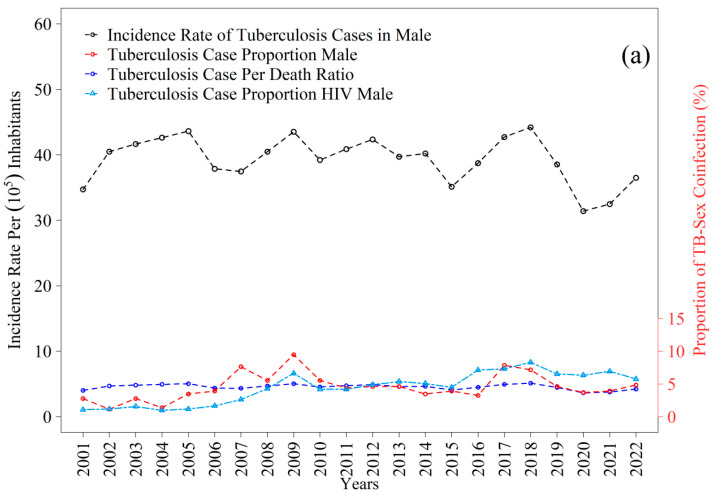

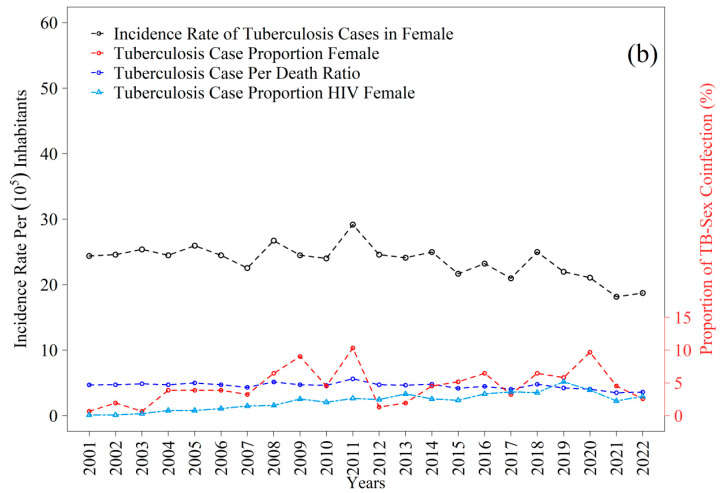
Incidence rate and proportion of tuberculosis (TB) cases from 2001 to 2022 in the 1st Health Region of Alagoas: (**a**) male and (**b**) female. Source: Authors, 2025.

The tuberculosis incidence time series reveal distinct patterns between men and women throughout the period examined (Figure 8a,b). Among men, the mean annual incidence was 39.29 cases per one hundred thousand inhabitants, with fluctuations that remained consistently above the mean in 2002–2005, 2008–2009, 2011–2014, 2017, and 2018. The highest value was recorded in 2018, at 44.22 per one hundred thousand, whereas the lowest occurred in 2020, at 31.41 per one hundred thousand. For women, the mean incidence was 23.66 per one hundred thousand, with periods exceeding the mean between 2001–2006, 2008–2014, and in 2018; the peak was observed in 2003, at 29.17 per one hundred thousand, and the lowest value in 2022, at 18.15 per one hundred thousand. Taken together, these trajectories reflect gradual declines consistent with those observed in the state, where rates decreased from thirty-one to eighteen per one hundred thousand inhabitants between 2012 and 2021, although current levels remain above the thresholds projected for elimination, as indicated in recent estimates [12,43].

The trajectories of TB–HIV coinfection exhibited broadly similar patterns between sexes, albeit with differing magnitudes across the years analyzed (Figure 8a,b). Among men, the proportion of tuberculosis cases with HIV showed a gradual increase from values near one percent at the beginning of the series, reaching between five and eight percent after 2015, with small peaks between 2017 and 2019. For women, the proportions also originated from very low levels, not exceeding one percent, and increased progressively to approximately four to six percent by the end of the series, with greater stability after 2016. Although both curves indicate a temporal rise in coinfection, the proportions remained systematically higher among men, reinforcing the nationally observed epidemiological pattern and underscoring the importance of systematic HIV screening in all tuberculosis cases. These trends parallel the gradual reductions in overall incidence but simultaneously reveal a persistent burden of coinfection whose magnitude poses additional challenges for disease control within the state.

**Figure 9:**
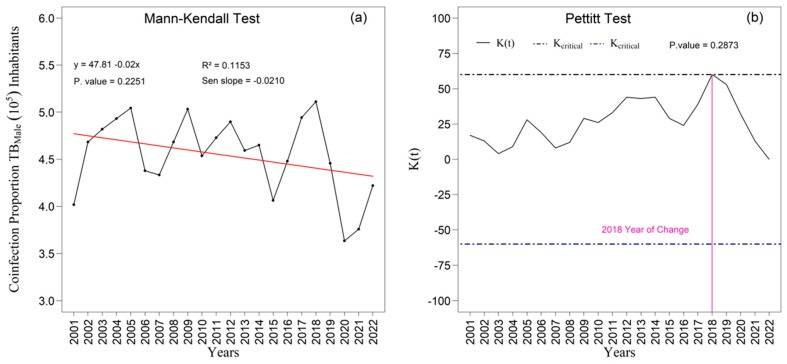
Temporal evolution of tuberculosis (TB) cases among males from 2001 to 2022 in the 1st Health Region of Alagoas: (**a**) Mann–Kendall trend test with Sen’s slope applied to the series, where the black line represents the observed values and the red line indicates the fitted linear trend; and (**b**) Pettitt change point test, in which the solid black curve represents the K(t) statistic, the dashed lines correspond to the critical thresholds, and the vertical magenta line indicates the year of abrupt chang. Source: Authors, 2025.

**Figure 10:**
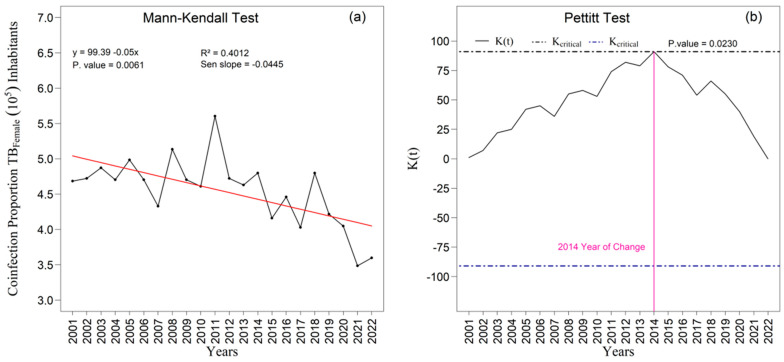
Temporal evolution of tuberculosis (TB) cases among females from 2001 to 2022 in the 1st Health Region of Alagoas: (**a**) Mann–Kendall trend test with Sen’s slope applied to the series, where the black line represents the observed values and the red line indicates the fitted linear trend; and (**b**) Pettitt change point test, in which the solid black curve represents the K(t) statistic, the dashed lines correspond to the critical thresholds, and the vertical magenta line indicates the year of abrupt change. Source: Authors, 2025.

The Figure 9a,b and Figure 10a,b present the temporal trends in tuberculosis rates among male and female individuals as assessed by the Mann–Kendall test, complemented by Sen’s slope estimator. For the male population (Figure 9a,b), the results did not indicate a statistically significant trend over the study period (z = −1.213; *p* = 0.2251). Although Sen’s slope suggested an average annual reduction of −0.02010 percentage points (95 percent confidence interval: −0.0487 to 0.0129), the confidence interval encompassing zero confirms the absence of robust evidence for a monotonic change in the male rates. In contrast, a statistically significant negative trend was observed among women (Figure 10a), with a Mann–Kendall statistic of z = −2.7414 (*p* = 0.0061). Sen’s slope showed an average annual decline of −0.0445 percentage points (95 percent confidence interval: −0.0749 to −0.0140), indicating a consistent and statistically supported reduction throughout the historical series; the entirely negative confidence interval reinforces the presence of a genuine downward trend in the female rate.

These findings show that while the male series fluctuates without a defined trend, the female series exhibits a continuous and statistically significant decline. The divergence observed in 2014 underscores the need for a gender-sensitive interpretation, since differences in access to health services, health-seeking behavior, occupational exposure, and surveillance sensitivity may explain this asymmetry. These contrasts reflect broader structural inequalities that shape detection, care, and outcomes in socioeconomically vulnerable regions. Sex-disaggregated trends therefore reveal both epidemiological differences and the structural mechanisms that unequally influence the course of tuberculosis among men and women.

The linear adjustment yielded R^2^ values of 0.12 percent for men (Figure 9a) and 0.40 percent for women (Figure 10a), suggesting slightly greater temporal coherence in the female series. The Pettitt test identified statistically significant change points in 2014 and 2018 at the five percent significance level (Figure 9b and Figure 10b). In 2014, the proportion among women rose to 0.70 (*p* = 0.2873), whereas the proportion among men declined to 0.62 (*p* = 0.0231), revealing sex-specific structural shifts. In 2018, a second break coincided with a peak in male incidence and with one of the instances of variance heterogeneity detected by Bartlett’s test, suggesting concomitant changes in surveillance practices, diagnostic intensity, or case composition.

In summary, the series is characterized by predominant normality and homogeneity of variance (Figure 3 and Figure 4), male predominance in case counts (Tables S1 and S2), greater dispersion and amplitude among men than among women (Tables S1 and S2; Figure 4), and mean incidence levels of 39.29 for men and 23.66 for women per one hundred thousand inhabitants, with sex-specific peaks and troughs that correspond to contextual shocks (Figure 8a,b). Within this framework, the exceptions are epidemiologically meaningful: non-normality among women in 2014, heteroscedasticity in 2005, 2016, and 2018, asymmetric fatality peaks between sexes, and structural breaks in 2014 and 2018 in the proportions of coinfection (Figure 9 and Figure 10). Together with the sociospatial gradient documented in Table 1 and Figure 3, and with the broader state-level context [29], these findings indicate that localized vulnerabilities in service coverage, diagnostic access, and broader social determinants continue to shape the dynamics of tuberculosis within the First Health Region of Alagoas.

**Figure 11:**
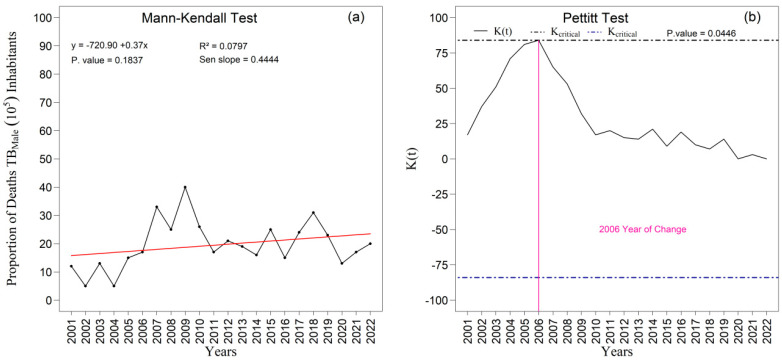
Temporal evolution of tuberculosis (TB) deaths among males from 2001 to 2022 in the 1st Health Region of Alagoas: (**a**) Mann–Kendall trend test with Sen’s slope applied to the series, where the black line represents the observed values and the red line indicates the fitted linear trend; and (**b**) Pettitt change point test, in which the solid black curve represents the K(t) statistic, the dashed lines correspond to the critical thresholds, and the vertical magenta line indicates the year of abrupt change. Source: Authors, 2025.

The temporal analysis of the proportions of tuberculosis-related deaths revealed distinct patterns between men and women (Figure 11) throughout the period 2001–2022 in the First Health Region of Alagoas. Among men, a slight upward tendency was observed, with a Sen’s slope of 0.4444, although this increase was not statistically significant (*p* = 0.1837), as indicated by the Mann–Kendall test. Even so, the series displayed marked fluctuations, particularly between 2006 and 2009, culminating in a structural change detected by the Pettitt test in 2006 (*p* = 0.0446), suggesting an inflection in the temporal pattern of male mortality. In contrast, the proportion of female deaths remained more stable over the two decades, with less pronounced variations and no indication of a statistically significant change point, reinforcing that the dynamics of tuberculosis mortality are considerably more volatile among men. When considered together, these findings reveal not only the persistent male excess mortality but also a notable inflection in the mid-2000s, possibly associated with gender-specific factors such as differences in occupational exposure, social vulnerability, and patterns of health-seeking behavior and access to diagnostic and clinical care.

The temporal analysis of the proportions of tuberculosis-related deaths in the First Health Region of Alagoas between 2001 and 2022 revealed contrasting patterns by sex. Among men (Figure 11a,b), a slight upward tendency in the proportion of deaths was observed, with a Sen’s slope of 0.4444, although this increase was not statistically significant (*p* = 0.1837), as indicated by the Mann–Kendall test. Nevertheless, the series exhibited pronounced fluctuations, particularly between 2006 and 2009, a period that coincided with the identification of a change point by the Pettitt test in 2006 (*p* = 0.0446). This result indicates the occurrence of a structural inflection in the temporal trajectory of male tuberculosis mortality, underscoring the greater instability of this indicator within the male population.

**Figure 12:**
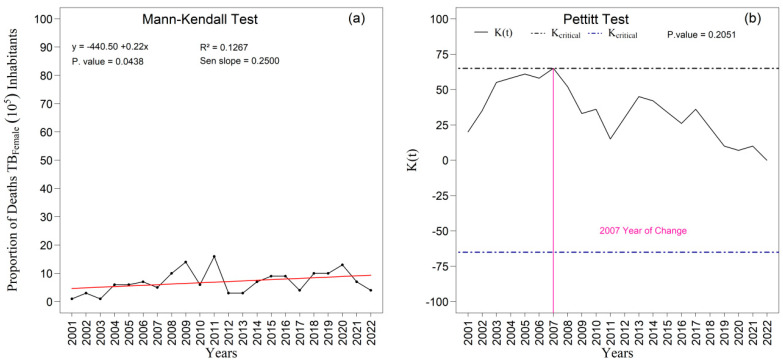
Temporal evolution of tuberculosis (TB) deaths among females from 2001 to 2022 in the 1st Health Region of Alagoas: (**a**) Mann–Kendall trend test with Sen’s slope applied to the series, where the black line represents the observed values and the red line indicates the fitted linear trend; and (**b**) Pettitt change point test, in which the solid black curve represents the K(t) statistic, the dashed lines correspond to the critical thresholds, and the vertical magenta line indicates the year of abrupt change. Source: Authors, 2025.

Among women, the pattern of the series initially appeared more stable, with no indication of abrupt changes over the period. However, the specific analysis of the proportion of female deaths (Figure 12a,b) revealed a statistically significant upward trend, as demonstrated by the Mann–Kendall test (*p* = 0.044; Sen’s slope = 0.250). The linear tendency suggests a consistent average annual increase throughout the two decades examined. The Pettitt test identified a potential change point in 2007, although without statistical significance (*p* = 0.205), indicating only a moderate alteration in the temporal pattern. Taken together, these findings demonstrate the persistent male excess mortality and the structural differences in the temporal pattern of tuberculosis mortality between sexes. The greater volatility observed among men may reflect inequalities related to occupational exposure, social vulnerability, and patterns of health-service utilization, whereas the gradual increase among women highlights persistent challenges in tuberculosis control that require sustained attention within public health surveillance and care policies.

**Figure 13:**
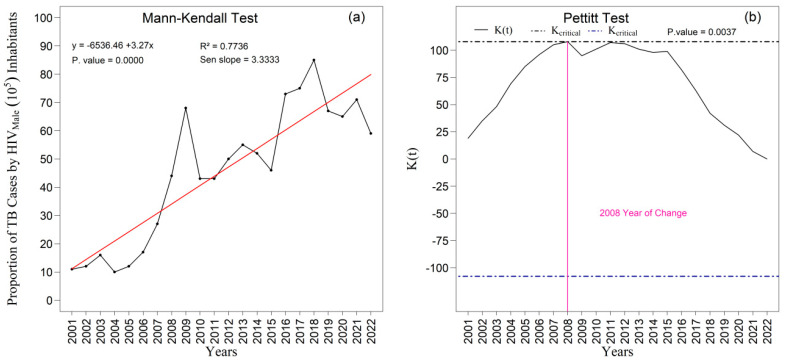
Temporal evolution of new tuberculosis (TB) cases associated with HIV among males from 2001 to 2022 in the 1st Health Region of Alagoas: (**a**) Mann–Kendall trend test with Sen’s slope applied to the series, where the black line represents the observed values and the red line indicates the fitted linear trend; and (**b**) Pettitt change point test, in which the solid black curve represents the K(t) statistic, the dashed lines correspond to the critical thresholds, and the vertical magenta line indicates the year of abrupt change. Source: Authors, 2025.

The analyses performed using the Mann–Kendall test for the proportion of TB/HIV coinfection among men (Figure 13a,b) revealed a highly significant upward trend (*p* < 0.000). The slope estimated by the Sen method (Sen’s slope = 3.33) indicates a pronounced average annual increase in the time series. Additionally, the Pettitt test corroborated the presence of this alteration by identifying an abrupt and statistically significant change point in 2008 (*p* = 0.004), signaling a relevant structural modification in the temporal pattern of coinfection beginning in that year.

**Figure 14:**
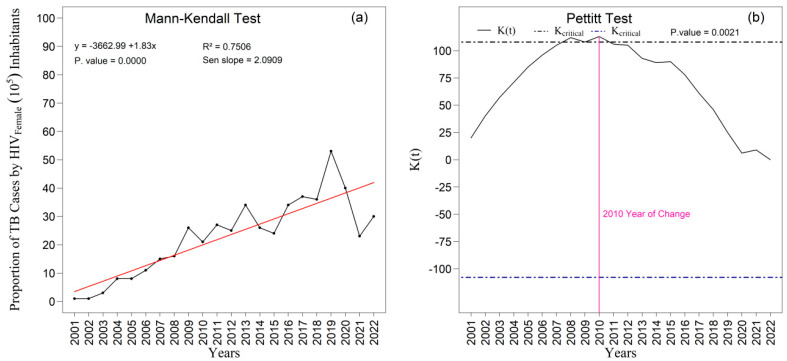
Temporal evolution of new tuberculosis (TB) cases associated with HIV among females from 2001 to 2022 in the 1st Health Region of Alagoas: (**a**) Mann–Kendall trend test with Sen’s slope applied to the series, where the black line represents the observed values and the red line indicates the fitted linear trend; and (**b**) Pettitt change point test, in which the solid black curve represents the K(t) statistic, the dashed lines correspond to the critical thresholds, and the vertical magenta line indicates the year of abrupt change. Source: Authors, 2025.

The Mann–Kendall test (Figure 14a) revealed a highly significant increasing trend in the proportion of TB/HIV coinfection cases among women (*p* < 0.000). The slope estimated by Sen’s method (Sen’s slope = 2.091) indicates a consistent average annual increase in the temporal series. The linear regression model exhibited substantial explanatory power (R^2^ = 0.751), reinforcing the coherence of the observed trend. In addition, the Pettitt test (Figure 14b) identified an abrupt and statistically significant change point in 2010 (*p* = 0.002), characterizing a relevant structural alteration in the temporal pattern of coinfection from that period onward.

## 4. Discussion

The analysis of the male (PCTB_MASC) and female (PCTB_FEMILE) tuberculosis time series from 2001 to 2022 identified distinct structural patterns in temporal dynamics and in the adequacy of statistical models, in accordance with national and international epidemiological evidence concerning sex-related differences in tuberculosis [1,2,3,5,19]. The male series exhibited significant positive autocorrelation (Durbin–Watson = 1.178; *p* = 0.010), confirmed by the persistence of autocorrelations in the autocorrelation function, indicating strong temporal dependence. In contrast, the female series showed no evidence of autocorrelation (Durbin–Watson = 1.669; *p* = 0.149), suggesting greater independence among consecutive years.

Both series demonstrated an overall downward trend, although with different magnitudes, a pattern already documented in long-term Brazilian analyses [7,12,15]. Among men, Sen’s slope was negative but non-significant (−0.021; *p* = 0.225). Among women, the negative trend was statistically significant (−0.045; *p* = 0.006), indicating a more rapid and consistent decline over the period, an interpretation aligned with literature highlighting sex-differentiated responses, treatment adherence, and access to primary healthcare services [3,5,19,20,49].

The presence of autocorrelation in the male series renders classical linear models inappropriate without corrective procedures, reinforcing the need for methods such as Prais–Winsten, Cochrane–Orcutt, or Newey–West robust estimators, as recommended in time-series analyses in epidemiological surveillance [7,19]. For the female series, the simple linear model remained adequate for inferential purposes. From an epidemiological perspective, the greater temporal persistence observed among men may reflect prolonged exposure to adverse social determinants and access barriers historically described for this group [5,19,20].

The notification data revealed that 14,197 new tuberculosis cases occurred in the First Health Region of Alagoas between 2001 and 2022, with a predominance among men (62.41 percent), a pattern similar to that observed in Brazil and globally [1,2,3]. The male series exhibited greater variability, higher interquartile ranges, and a larger presence of extreme values, as well as oscillations associated with external events, such as the COVID-19 pandemic in 2020, which affected tuberculosis diagnosis nationwide [50,51]. The female series demonstrated lower variability, a more homogeneous distribution, and fewer atypical observations.

Incidence rates reinforce these differences: among men, the mean annual incidence was 39.29 per one hundred thousand inhabitants, with a peak in 2018; among women, the mean was 23.66 per one hundred thousand, with a peak in 2003. These levels remain above the elimination targets proposed by the World Health Organization [1], reflecting sociospatial inequalities that persist in Brazil.

TB–HIV coinfection increased in both sexes, particularly among men, reaching proportions of five to eight percent after 2015. These findings are consistent with emerging epidemiological analyses showing gender-specific vulnerabilities in TB–HIV coinfection and treatment outcomes [49]. Among women, proportions ranged from four to six percent. These findings are consistent with national and global stabilization followed by resurgence of coinfection in vulnerable populations [2,15,16]. Such results underscore the need for universal testing, in accordance with WHO and Brazilian Ministry of Health guidelines [1,2].

Trend tests (Mann–Kendall) indicated the absence of significant trends among men (z = −1.213; *p* = 0.225), despite the estimated annual decline, whereas among women the trend was significant (z = −2.741; *p* = 0.006). The literature indicates that sex disparities reflect differences in exposure, social determinants, self-care practices, and access to healthcare networks [3,19,20].

The linear adjustment exhibited low explanatory power (R^2^ = 0.12 percent for men; 0.40 percent for women), consistent with the multifactorial nature of tuberculosis, whose incidence is modulated by poverty, household density, comorbidities, and broader social vulnerabilities [3,5,14,19]. The Pettitt test identified structural breaks in 2014 and 2018, possibly associated with reorganizations of surveillance policies, intensification of active case finding, or fluctuations related to broader health crises [2,12,19].

Complementary statistical analyses confirmed normality and homogeneity of variance throughout most of the period, with occasional exceptions including non-normality in 2014 and heteroscedasticity in 2005, 2016, and 2018. These findings are consistent with studies that document structural breaks and fluctuations in long epidemiological series due to external factors or operational changes within health systems [2,14,50].

Regarding outcomes, the proportions of tuberculosis-related deaths exhibited distinct patterns: among men, a non-significant increasing trend accompanied by high variability and a change point in 2006; among women, a statistically significant increasing trend (*p* = 0.044), albeit with lower instability. These results are compatible with findings that indicate higher lethality among women in specific contexts of diagnostic delay or reduced access to hospital care [20,51].

TB–HIV coinfection among women displayed a highly significant increasing trend (*p* < 0.000), with an annual mean increase of 2.09 percentage points and a structural break in 2010. This pattern reflects patterns previously documented in temporal analyses of HIV in Brazil and other developing countries [15,16,52].

## 5. Conclusions

The analysis of tuberculosis time series revealed substantial differences between men and women in the First Health Region of Alagoas from 2001 to 2022. The male series exhibited significant autocorrelation, indicating strong temporal dependence, whereas the female series did not display this pattern, suggesting greater independence across years. Both series showed an overall downward trajectory; however, only the female trend reached statistical significance, indicating a more consistent decline over the period, while the negative trend observed among men did not attain statistical significance.

Men accounted for the majority of reported cases and demonstrated greater annual variability, as well as heightened sensitivity to external events such as the COVID-19 pandemic. Incidence rates were also higher in the male group and remained above international elimination targets. TB–HIV coinfection increased in both sexes, with a more pronounced rise among men. Among women, a highly significant upward trend was identified, accompanied by a structural break beginning in 2010, indicating changes in detection and surveillance dynamics over time.

Trend and structural change tests identified breakpoints in 2014 and 2018, possibly related to adjustments in surveillance policies and broader public health events. The low explanatory power of simple linear models confirms that tuberculosis incidence is shaped by multiple social, economic, and healthcare-related determinants. Mortality analyses revealed a non-significant increasing trend among men and a significant increase among women, the latter with lower temporal instability. These findings underscore sex-specific vulnerabilities and demonstrate the need for differentiated approaches to prevention, testing, and care.

In summary, the results indicate that tuberculosis remains a significant public health challenge in the region. The identified sex differences highlight the need for tailored strategies in prevention, diagnostic outreach, and clinical management, as well as the strengthening of epidemiological surveillance and primary healthcare actions aimed at reducing incidence, coinfection, and adverse outcomes associated with the disease.

## Figures and Tables

**Table 1:** Geographical parameters in the 1st Health Region of the state of Alagoas for the period from 2001 to 2022.

ID	Municipality	Total Population	Population Percentage	Population Density (Inhab·km^−2^)	Municipal Human Development Index (MHDI)
1	Barra de Santo Antônio	16.365	1.338	124.58	0.557
2	Barra de São Miguel	7.944	0.649	106.99	0.615
3	Coqueiro Seco	5.581	0.456	140.91	0.586
4	Flexeiras	9.618	0.786	28.82	0.527
5	Maceió	957.916	78.308	1880.77	0.721
6	Marechal Deodoro	60.370	4.935	177.05	0.642
7	Messias	15.405	1.259	134.95	0.568
8	Paripueira	13.835	1.131	149.10	0.605
9	Pilar	35.370	2.891	136.24	0.610
10	Rio Largo	93.927	7.678	319.68	0.643
11	Santa Luzia do Norte	6.919	0.566	239.77	0.597
12	Satuba	24.278	0.002	588.30	0.660

Source: Authors, 2025.

**Table 2:** Durbin–Watson test results for annual male and female tuberculosis (TB) case series in the First Health Region of Alagoas for the period 2001–2022.

Data Series	DW	*p*-Value	Analytical Interpretation
PCTB_MALE	1.178	0.010	Statistically significant positive autocorrelation
PCTB_FEMALE	1.669	0.148	No statistically significant autocorrelation is present

Source: Authors, 2025.

## Data Availability

The data supporting the findings of this study are available from the corresponding author upon reasonable request.

## Supplementary Materials

The following supporting information can be downloaded at: https://www.mdpi.com/article/10.3390/ijerph22121846/s1, Equation (S1): Proportion of new tuberculosis cases by sex (%TB/sex); Equation (S2): Sex-specific tuberculosis incidence rate (TITB/sex); Equation (S3): Sign Function for the Mann–Kendall Test; Equation (S4): Expected Value of the Mann–Kendall Statistic; Equation (S5): Variance of the Mann–Kendall Statistic; Equation (S6): Tie-Adjusted Variance of the Mann–Kendall Statistic; Equation (S7): Standardized Z-Statistic for the Mann–Kendall Test; Equation (S8): Number of Pairwise Slopes for the Sen Estimator; Equation (S9): Definition of the Pettitt Test Statistic; Table S1: Statistical parameters of new male TB cases in the 1st Health Region of Alagoas (2001–2022); Table S2: Statistical parameters of new female TB cases in the 1st Health Region of Alagoas (2001–2022).

## Abbreviations

1st HRA	First Health Region of Alagoas
AIDS	Acquired Immunodeficiency Syndrome
B_0_	Bartlett Test Statistic
CAPES	Coordination for the Improvement of Higher Education Personnel
CNPq	National Council for Scientific and Technological Development
CV	Coefficient of Variation
DATASUS	Department of Informatics of the Unified Health System
HDI	Human Development Index
IBGE	Brazilian Institute of Geography and Statistics
IQR	Interquartile Range
LL	Lower Limit
MHDI	Municipal Human Development Index
MK	Mann–Kendall Test
MS	Ministry of Health (Brazil)
NC	New Cases
NS	Not Significant
PDJ	Junior Postdoctoral Fellowship
PHC	Primary Healthcare
PNPD	National Postdoctoral Program
PT	Pettitt Test
Q1	First Quartile
Q3	Third Quartile
R^2^	Coefficient of Determination
SD	Standard Deviation
SEN	Sen’s Slope Estimator
SINAN	Notifiable Diseases Information System
SW	Shapiro—Wilk Test
SUS	Unified Health System (Brazil)
TB	Tuberculosis
TB–HIV	Tuberculosis–Human Immunodeficiency Virus Coinfection
TR	Total Range
UFAC	Federal University of Acre
UFAL	Federal University of Alagoas
UFF	Fluminense Federal University
UL	Upper Limit
WHO	World Health Organization

## References

[1] Sá L.C.B.d., Meirelles R.C., Atherino C.C.T., Fernandes J.R.C., Ferraz F.R. (2007). Pharyngolaryngeal Tuberculosis. Rev. Bras. Otorrinolaringol..

[2] Cavalcante E.F.O., Silva D.M.G.V.S. (2013). Epidemiological Profile of Individuals with Tuberculosis. Rev. Rene.

[3] Reis D.C.d., Almeida T.A.C.d., Quites H.F.d.O., Sampaio M.M. (2013). Epidemiological Profile of Tuberculosis in the City of Belo Horizonte (MG), from 2002 to 2008. Rev. Bras. Epidemiol..

[4] Mendes M.d.S., Oliveira A.L.S.d., Pimentel L.M.L.M., Figueiredo T.M.R.M.d., Schindler H.C. (2021). Spatial Analysis of Tuberculosis among Children under 15 Years of Age and Socioeconomic Risk: An Ecological Study in Paraíba, 2007–2016. Epidemiol. E Serviços Saúde.

[5] Bortoluci A.B., Silveira Tomiazzi J., Amaral Carrasco R., Paula Marques Ramos A., Vinicius Pimenta Rodrigues M. (2016). Evaluation of the Spatial Distribution of Tuberculosis in the Pontal Do Paranapanema Region. Colloq. VITAE.

[6] Bezerra Gama Nilo M.C. (2018). Social Network Analysis as a Strategy for Evaluating Health Programs for Tuberculosis Control. Redes. Rev. Hisp. Para Análisis Redes Soc..

[7] Souza N.K.M.d., Machado L.D.S., Alves D.F., Silva Filho L.A.d., Silva V.M.d. (2025). Persistência Temporal e Espacial de Casos de Tuberculose Nos Municípios Brasileiros Entre 2001 e 2022. Cien. Saude Colet..

[8] Cicchelero L.M., Leandro G.C.W., Andrade L.d., Meneguello J.E., Caleffi-Ferracioli K.R., Cardoso R.F., Scodro R.B.d.L. (2025). From Patterns to Projections: A Spatiotemporal Distribution of Drug-Resistant Tuberculosis in Paraná, Brazil (2012–2023). Pathogens.

[9] Barbosa I.R., Henrique G.L. (2014). Characterization of Tuberculosis Cases in a Priority Municipality in the State of Rio Grande Do Norte. Rev. APS.

[10] Tavares C.M., Cunha A.M.S.d., Gomes N.M.C., Lima A.B.d.A., Santos I.M.R.d., Acácio M.d.S., Santos D.M.d., Souza C.D.F.d. (2020). Trend and Epidemiological Characterization of Tuberculosis in Alagoas, 2007–2016. Cad. Saude Colet..

[11] Villalva-Serra K., Barreto-Duarte B., Miguez-Pinto J.P., Queiroz A.T.L., Rodrigues M.M., Rebeiro P.F., Amorim G., Cordeiro-Santos M., Sterling T.R., Araújo-Pereira M. (2024). Impact of Xpert MTB/RIF Implementation in Tuberculosis Case Detection and Control in Brazil: A Nationwide Intervention Time-Series Analysis (2011–2022). Lancet Reg. Health Am..

[12] Lima L.V.d., Pavinati G., Oliveira R.R.d., Couto R.d.M., Alves K.B.A., Magnabosco G.T. (2024). Temporal Trend in the Incidence of Tuberculosis-HIV Coinfection in Brazil, by Macro-Region, Federative Unit, Sex and Age Group, 2010-2021. Epidemiol. Serviços Saúde.

[13] Santos B.A., Ribeiro C.J.N., Santos A.D.d., Sousa Á.F.L.d., Siqueira T.S., Andrade L.A., Santos A.J.d., Lima S.V.M.A. (2024). Surveillance of TB-HIV Coinfection in Brazil: A Space-Time Approach. Rev. Bras. Epidemiol..

[14] World Health Organization (WHO) (2021). Global Tuberculosis Report 2021.

[15] Cavalin R.F., Pellini A.C.G., Lemos R.R.G.d., Sato A.P.S. (2020). TB-HIV Coinfection. Rev. Saude Publica.

[16] Macedo L.R., Maciel E.L.N., Struchiner C.J. (2021). Vulnerable Populations and the Outcome of Tuberculosis Cases in Brazil. Cien. Saude Colet..

[17] Oliveira e Silva H., Gonçalves M.L.C. (2009). Health Risk Behaviors among High School Adolescents. Rev. Bras. Promoção Saúde.

[18] Santos Júnior C.J.d., Rocha T.J.M., Soares V.d.L. (2019). Temporal Analysis of Tuberculosis–HIV Coinfection Cases in the Population of a Northeastern Brazilian State. Rev. Epidemiol. Controle Infecção.

[19] Netto A.R. (1999). Impact of Health Sector Reform on Tuberculosis Services in Brazil. Bol. Pneumol. Sanitária.

[20] Villa T.C.S., Brunello M.E.F., Andrade R.L.d.P., Orfão N.H., Monroe A.A., Nogueira J.d.A., Silva-Sobrinho R.A.d., Pinto E.S.G., Vendramini S.H.d.F., Scatena L.M. (2018). Primary Health Care Management Capacity for Tuberculosis Control in Different Regions of Brazil. Texto Contexto Enferm..

[21] Muniz J.N., Palha P.F., Monroe A.A., Gonzales R.C., Ruffino Netto A., Villa T.C.S. (2005). The Incorporation of Active Case Finding of Respiratory Symptomatic Individuals for Tuberculosis Control in the Practice of Community Health Workers. Cien. Saude Colet..

[22] Mann H.B. (1945). Nonparametric Tests Against Trend. Econometrica.

[23] Pettitt A.N. (1979). A Non-Parametric Approach to the Change-Point Problem. Appl. Stat..

[24] State of Alagoas (2020). Decree No. 72,438 of December 22, 2020. Establishes the Health Administrative Regions of the State of Alagoas.

[25] Yue S., Pilon P., Cavadias G. (2002). Power of the Mann–Kendall and Spearman’s Rho Tests for Detecting Monotonic Trends in Hydrological Series. J. Hydrol..

[26] Helsel D.R., Hirsch R.M. (2002). Statistical Methods in Water Resources.

[27] Hamed K.H. (2008). Trend Detection in Hydrologic Data: The Mann–Kendall Trend Test under the Scaling Hypothesis. J. Hydrol..

[28] Hamed K.H., Ramachandra Rao A. (1998). A Modified Mann-Kendall Trend Test for Autocorrelated Data. J. Hydrol..

[29] Instituto Brasileiro de Geografia e Estatística (IBGE) Censo Demográfico 2022: Resultados Preliminares. https://www.ibge.gov.br/censo.

[30] Snedecor G.W., Cochran W.G. (1989). Statistical Methods.

[31] Ghasemi A., Zahediasl S. (2012). Normality Tests for Statistical Analysis: A Guide for Non-Statisticians. Int. J. Endocrinol. Metab..

[32] Brasil. Ministério da Saúde—DATASUS Sistema de Informação de Agravos de Notificação (SINAN)—Tuberculose. http://tabnet.datasus.gov.br/cgi/tabcgi.exe?sinannet/cnv/tubercal.def.

[33] R Core Team (2024). R: A Language and Environment for Statistical Computing.

[34] Gois G.d., Terassi P.M.d.B., Moreira J.G.d.V., Freitas J.d.S., Sobral B.S., Muniz M.A., Costa Júnior D.S.d., Aleluia I.S.S., Vanderlei M.H.G.d.S., Carvalho Neto G.d. (2024). Índice de Anomalia de Chuva e Sua Associação Ao El Niño–Oscilação Sul (ENOS) Em Rio Branco (AC), Brasil. Caminhos Geogr..

[35] Shapiro S.S., Wilk M.B. (1965). An Analysis of Variance Test for Normality (Complete Samples). Biometrika.

[36] Schlotzhauer S.D., Littell R.C. (1997). SAS System for Elementary Statistical Analysis.

[37] Bartlett M.S. (1937). Properties of Sufficiency and Statistical Tests. Proc. R. Soc. Lond. A Math. Phys. Sci..

[38] Kendall M.G. (1949). Rank and Product-Moment Correlation. Biometrika.

[39] Almeida H.A.d., Bezerra Júnior J. (2015). Seasonal and Interdecadal Variability of Rainfall in the Geographic Microregions of the State of Paraíba. Rev. Bras. Geogr. Física.

[40] Moreira J.G.d.V., Naghettini M. (2016). Detection of Temporal Monotonic Trends and Relationship with Type I and II Errors: A Case Study of Annual Maximum Daily Rainfall Series in the State of Acre. Rev. Bras. Meteorol..

[41] Penereiro J.C., Badinger A., Maccheri N.A., Meschiatti M.C. (2018). Seasonal Trend Distributions of Mean Temperature and Precipitation in Brazilian Biomes. Rev. Bras. Meteorol..

[42] Cabral Júnior J.B., Lucena R.L. (2020). Analysis of Precipitations by Non-Parametric Tests of Mann-Kendall and Kruskal-Wallis. Mercator.

[43] Brito M.I.B.d.S., Oliveira E.C.A.d., Barbosa C.S., Gomes E.C.d.S. (2023). Factors Associated with Severe Forms and Deaths from Schistosomiasis and Application of Probabilistic Linkage in Databases, Pernambuco, 2007–2017. Rev. Bras. Epidemiol..

[44] Chebana F., Ouarda T.B.M.J., Duong T.C. (2013). Testing for Multivariate Trends in Hydrologic Frequency Analysis. J. Hydrol..

[45] de Azevedo J.V.V., dos Santos C.A.C., Tavares Silva M., Alves de Olinda R., Aparecida da Silva Santos D. (2017). Analysis of Climatic Variations in the Occurrence of Respiratory Diseases Caused by Influenza among the Elderly in the Metropolitan Region of João Pessoa-PB. Soc. Nat..

[46] Piovezan R., Visockas A., de Azevedo T.S., Von Zuben C.J., Sallum M.A.M. (2019). Spatial–Temporal Distribution of Aedes (Stegomyia) Aegypti and Locations of Recycling Units in Southeastern Brazil. Parasit Vectors.

[47] Mandú T.B., Dos A.C., Gomes S., Souza R., Vale D. (2019). Association between Heat Index and Hospitalizations for Acute Myocardial Infarction in Manaus-AM. Hygeia.

[48] Bari S.H., Rahman M.T.U., Hoque M.A., Hussain M.d.M. (2016). Analysis of Seasonal and Annual Rainfall Trends in the Northern Region of Bangladesh. Atmos. Res..

[49] Al-Rajhi A.T., Alqassim A.Y. (2025). Perceived Stigma and Associated Factors Among Patients with Tuberculosis and Their Families in Jazan Region, Saudi Arabia. Healthcare.

[50] Goes Da Silva E., Decele J., Vieira S., Lima Cavalcante A., Gabrielly L., De M., Santos L., Rebelo A.P., Rodrigues A., Carolina T. (2015). Epidemiological Profile of Tuberculosis in the State of Alagoas, 2007–2012. Cad. Grad.-Ciências Biológicas E Saúde-UNIT-ALAGOAS.

[51] Tavares R.B.V., Gomes D., Berra T.Z., Alves Y.M., Ramos A.C.V., Popolin M.A.P., Abade A.d.S., Zini N., Tártaro A.F., Alves J.D. (2025). The Temporal Trends of Mortality Due to Tuberculosis in Brazil: Tracing the Coronavirus Disease 2019 (COVID-19) Pandemic’s Effect Through a Bayesian Approach and Unmasking Disparities. Microorganisms.

[52] Carvalho L.P., Shibata L.H., Freitas M.C., Costa S.C.d., Novais Júnior R.T., Milhomem L.M.A., Cunha T.R., Quaresma P.V.C. (2020). Overview of Pulmonary Tuberculosis in Priority Municipalities of the State of Pará, Brazil, 2013–2017. Braz. J. Health Rev..

