# Supplementary material for: Temporal Trends in Tuberculosis Incidence in the 1st Health Region of Alagoas, Brazil (2001–2022)
Source: Int J Environ Res Public Health. 2025 Dec 10;22(12):1846. doi: 10.3390/ijerph22121846 (PMC12732565; doi:10.3390/ijerph22121846)
Supplement: Supplementary file 1 [file ijerph-22-01846-s001.zip › ijerph-3973119-supplementary.pdf]

Supplementary Material

Equation S1. Proportion of new tuberculosis cases by sex (%TB/sex)

$$\%TB_{Sex} = \frac{\text{Number of new TB cases by sex}}{\text{Total number of TB cases in the year}} \text{ x } 10^2 \quad \text{(S1)}$$

Equation S2. Sex-specific tuberculosis incidence rate (TITB/sex)

$$TI_{TB/Sex} = \frac{\text{Number of new TB cases by sex}}{\text{Total population by sex in the year}} \text{ x}10^5 \quad \text{(S2)}$$

Equation S3. Sign Function for the Mann–Kendall Test

$$S = \begin{cases} +1 \; if \; (x_j - x_i) > 0 \\ 0 \; if \; (x_j - x_i) = 0 \\ -1 \; if \; (x_j - x_i) < 0 \end{cases} \quad \text{(S3)}$$

Equation S4. Expected Value of the Mann–Kendall Statistic

$$\mathrm{E(s)} = 0 \quad \text{(S4)}$$

Equation S5. Variance of the Mann–Kendall Statistic

$$Var(s) = \frac{[n(n-1)(2n+5)]}{18} \quad \text{(S5)}$$

Equation S6. Tie-Adjusted Variance of the Mann–Kendall Statistic

$$Var(s) = \frac{1\left[n(n-1)(2n+5) - \sum_{p=1}^{q} t_p \, (t_p - 1)(2t_{p+5})\right]}{18} \quad \text{(S6)}$$

Where: t_p represents the number of tied observations in the p-th group, and q is the total number of groups with ties.

Equation S7. Standardized Z-Statistic for the Mann–Kendall Test

$$Z_{MK} = \begin{cases} \frac{S-1}{\sqrt{Var\,(S)}} \; ; if \; S > 0 \\ 0 \; ; if \; S = 0 \\ \frac{S+1}{\sqrt{Var\,(S)}} ; if \; S < 0 \end{cases} \quad \text{(S7)}$$

Equation S8. Number of Pairwise Slopes for the Sen Estimator

$$N = \frac{n(n-1)}{2} \quad \text{(S8)}$$

Equation S9. Definition of the Pettitt Test Statistic

$$k(t) = Max_{1 \le t \le T} \left| U_{t,T} \right| \quad \text{(S9)}$$

Table S1. Statistical parameters of new male TB cases (NC – New Cases): percentage (%) representing the proportion of cases in relation to the total number recorded throughout the entire time series, mean ($\bar{x}$), median (Md), minimum (Min) and maximum (Max) values, lower (LL) and upper (UL) limits, total range (TR), coefficient of variation (CV %), skewness (Sk) and kurtosis (K), standard deviation (SD), lower (Q1) and upper (Q3) quartiles, and interquartile range (IQR) in the 1st Health Region of Alagoas for the period from 2001 to 2022.

Year	NC	%	$\bar{x}$	M_d	Values		Limits	
					M_{in}	M_{ax}	UL	LL
2001	356	4.02	29.67	30.00	*12*	43	18.38	41.38
2002	415	4.68	34.58	31.50	17	*63*	10.00	58.00
2003	427	4.82	35.58	35.00	14	55	13.00	59.00
2004	437	4.93	36.42	35.50	18	53	*1.38*	*76.38*
2005	*447*	5.04	37.25	37.00	26	55	*28.63*	45.63
2006	388	4.38	32.33	32.00	*24*	40	18.25	48.25
2007	384	4.33	32.00	31.50	22	46	21.00	41.00
2008	415	4.68	34.58	34.50	23	48	16.75	50.75
2009	446	5.03	37.17	38.00	27	51	14.38	57.38
2010	402	4.54	33.50	35.00	23	44	11.38	54.38
2011	419	4.73	34.92	34.00	28	45	18.00	50.00
2012	434	4.90	36.17	36.00	25	50	17.25	55.25
2013	407	4.59	33.92	34.00	25	41	22.75	44.75
2014	412	4.65	34.33	34.00	23	48	12.50	54.50
2015	360	4.06	30.00	32.00	19	43	12.13	47.13
2016	397	4.48	33.08	33.00	*24*	50	7.63	56.63
2017	438	4.94	36.50	39.50	*24*	44	17.00	57.00
2018	*453*	*5.11*	*37.75*	38.50	*24*	53	28.63	47.63
2019	395	4.46	32.92	34.50	19	44	11.38	56.38
2020	*322*	*3.63*	26.83	27.50	14	42	*-4.63*	58.38
2021	333	3.76	27.75	27.00	18	42	12.50	42.50
2022	374	4.22	31.17	30.50	22	47	17.00	43.00
**Total cases**	**8861**							

Year	TR	Coefficients			SD	Quartile		IQR
		CV (%)	S_K	K		Q_1	Q_3	
2001	31	27.09	+	Platykurtic	8.04	27.00	32.75	5.75
2002	46	36.38	+	Platykurtic	12.58	28.00	40.00	12.00
2003	41	*32.72*	+	Platykurtic	11.64	30.25	41.75	11.50
2004	35	*32.08*	+	Platykurtic	11.68	29.50	48.25	18.75
2005	29	19.43	+	Platykurtic	7.24	35.00	39.25	4.25
2006	16	16.01	+	Platykurtic	5.18	29.50	37.00	7.50
2007	24	19.31	+	Platykurtic	6.18	28.50	33.50	5.00
2008	25	20.72	+	Platykurtic	7.17	29.50	38.00	8.50
2009	24	20.42	-	Platykurtic	7.59	30.50	41.25	10.75
2010	21	20.11	-	Platykurtic	6.74	27.50	38.25	10.75
2011	17	15.37	+	Platykurtic	5.37	30.00	38.00	8.00
2012	25	20.72	+	Platykurtic	7.49	31.50	41.00	9.50
2013	16	12.79	+	Platykurtic	4.34	31.00	36.50	5.50
2014	25	23.71	+	Platykurtic	8.14	28.25	38.75	10.50
2015	24	22.20	+	Platykurtic	6.66	25.25	34.00	8.75

2016	26	23.61	+	Platykurtic	7.81	26.00	38.25	12.25
2017	20	19.32	+	Platykurtic	7.05	32.00	42.00	10.00
2018	29	20.15	-	Platykurtic	7.61	35.75	40.50	4.75
2019	25	25.27	-	Platykurtic	8.32	28.25	39.50	11.25
2020	28	*35.46*	-	Platykurtic	9.51	19.00	34.75	15.75
2021	24	23.81	+	Platykurtic	6.61	23.75	31.25	7.50
2022	25	22.14	+	Platykurtic	6.90	26.75	33.25	6.50

Source: Authors, 2025.

Table S2. Statistical parameters of new female TB cases (NC – New Cases): percentage (%) representing the proportion of cases in relation to the total number recorded throughout the entire time series, mean (x̅), median (Md), minimum (Min) and maximum (Max) values, lower (LL) and upper (UL) limits, total range (TR), coefficient of variation (CV %), skewness (Sk) and kurtosis (K), standard deviation (SD), lower (Q1) and upper (Q3) quartiles, and interquartile range (IQR) in the 1st Health Region of Alagoas for the period from 2001 to 2022.

Year	NC	%	$\bar{x}$	M_d	Values		Limits	
					M_{in}	M_{ax}	UL	LL
2001	250	4.69	20.83	21.50	11	34	6.63	33.63
2002	252	4.72	21.00	21.00	12	*38*	7.00	33.00
2003	260	4.87	21.67	23.00	11	32	*-2.38*	46.63
2004	251	4.70	20.92	21.00	9	32	8.00	34.00
2005	266	4.99	22.17	21.50	13	31	*-2.75*	47.25
2006	251	4.70	20.92	21.00	12	28	12.63	29.63
2007	231	4.33	19.25	18.50	12	29	*2.75*	34.75
2008	274	5.13	22.83	23.50	12	32	9.75	35.75
2009	251	4.70	20.92	19.50	16	30	13.75	27.75
2010	246	4.61	20.50	20.00	*6*	34	7.63	32.63
2011	*299*	*5.60*	*24.92*	24.50	18	30	10.50	40.50
2012	252	4.72	21.00	21.00	12	30	13.50	27.50
2013	247	4.63	20.58	21.00	13	27	8.50	32.50
2014	256	4.80	21.33	19.50	15	36	8.75	30.75
2015	222	4.16	18.50	19.50	11	24	9.38	28.38
2016	238	4.46	19.83	19.00	15	29	7.38	30.38
2017	215	4.03	17.92	18.00	13	24	9.00	27.00
2018	256	4.80	*21.33*	22.00	15	28	*15.63*	26.63
2019	225	4.22	18.75	18.00	13	27	12.13	25.13
2020	216	4.05	18.00	19.00	9	32	1.63	32.63
2021	*186*	*3.49*	15.50	15.50	*6*	21	9.50	23.50
2022	192	3.60	16.00	15.50	*6*	23	4.75	28.75
**Total cases**	**5336**							
**Year**	TR	**Coefficients**			SD	**Quartile**		IQR
		CV **(%)**	S_K	K		Q_1	Q_3	
2001	23	29.58	+	Platykurtic	6.16	16.75	23.50	6.75
2002	26	*31.72*	+	Platykurtic	6.66	16.75	23.25	6.50
2003	21	*33.13*	+	Platykurtic	7.18	16.00	28.25	12.25
2004	23	29.64	+	Platykurtic	6.20	17.75	24.25	6.50
2005	18	29.79	+	Platykurtic	6.60	16.00	28.50	12.50
2006	16	18.85	+	Platykurtic	3.94	19.00	23.25	4.25

2007	17	29.73	+	Platykurtic	5.72	14.75	22.75	8.00
2008	20	27.88	+	Platykurtic	6.37	19.50	26.00	6.50
2009	14	19.50	+	Platykurtic	4.08	19.00	22.50	3.50
2010	28	*32.72*	+	Platykurtic	6.71	17.00	23.25	6.25
2011	12	18.70	+	Platykurtic	4.66	21.75	29.25	7.50
2012	18	22.79	+	Platykurtic	4.79	18.75	22.25	3.50
2013	14	20.87	+	Platykurtic	4.29	17.50	23.50	6.00
2014	21	29.40	+	Platykurtic	6.27	17.00	22.50	5.50
2015	13	21.06	+	Platykurtic	3.90	16.50	21.25	4.75
2016	14	21.16	+	Platykurtic	4.20	16.00	21.75	5.75
2017	11	18.51	+	Platykurtic	3.32	15.75	20.25	4.50
2018	13	16.88	+	Platykurtic	3.60	19.75	22.50	2.75
2019	14	18.77	+	Platykurtic	3.52	17.00	20.25	3.25
2020	23	*36.24*	-	Platykurtic	6.52	13.25	21.00	7.75
2021	15	26.31	+	Platykurtic	4.08	14.75	18.25	3.50
2022	17	*30.62*	+	Platykurtic	4.90	13.75	19.75	6.00

Source: Authors, 2025.
